# Prestimulus Periodic and Aperiodic Neural Activity Shapes McGurk Perception

**DOI:** 10.1523/ENEURO.0431-24.2025

**Published:** 2025-10-10

**Authors:** Vinsea A. V. Singh, Vinodh G. Kumar, Arpan Banerjee, Dipanjan Roy

**Affiliations:** ^1^Cognitive Brain Dynamics Lab, National Brain Research Centre, Gurugram 122052, India; ^2^Department of Neurology, Penn State College of Medicine, Hershey, Pennsylvania 17033; ^3^Center for Brain Science and Applications, School of AIDE, Indian Institute of Technology, Jodhpur 342030, India

**Keywords:** aperiodic activity, AV speech perception, EEG, McGurk, logistic mixed-effects model, periodic power, prestimulus

## Abstract

Previous studies emphasize the importance of prestimulus neural oscillations in shaping endogenous brain states that substantially impact perceptual outcomes. However, what features in such oscillations drive perception remains unknown. Furthermore, research has shown that non-oscillatory activity is also important for cognitive processing. However, their interaction prior to perceiving a multisensory stimulus remains unexplored. In this human EEG study (*n* = 18, 10 males and 8 females), we investigated the role of prestimulus periodic power and aperiodic activity in modulating perception of the widely studied McGurk illusion on a trial-by-trial basis. Using logistic mixed-effects models, we reveal that the illusion perception is associated with reduced prestimulus alpha (8–12 Hz) and beta (15–30 Hz) power over frontal and occipital regions; increased theta (4–7 Hz) power in parietal, central, and occipital regions; and increased gamma (31–45 Hz) power across the scalp. Furthermore, lower aperiodic offset and exponent values in central, parietal, and occipital regions also predicted illusory responses. Our logistic mixed interaction models revealed that the aperiodic exponent and periodic power jointly influence the perception of upcoming McGurk stimuli. Specifically, a decrease in occipital theta and global beta power and an increase in occipital and parietal gamma power were associated with a steeper slope. We conclude that the predominant source of variations in the prestimulus state is the aperiodic activity and that fluctuations in both periodic and aperiodic activity account for inter-trial variability in the perception of the McGurk illusion.

## Significance Statement

Prestimulus brain oscillations and aperiodic activity are fundamental to understanding individual perceptual and cognitive processing during multisensory speech perception. However, during multisensory integration between auditory and visual streams, how periodic and aperiodic activity sculpts inter-individual and inter-trial differences in multisensory perception remains largely unknown. In this EEG study, we discovered that lower aperiodic offset and exponent (slope) values in central, parietal, and occipital regions predicted illusory responses. Using statistical interaction models, we further show that the mechanisms of susceptibility to illusory speech perception arise from the significant interplay between aperiodic background activity and oscillatory features. This interplay between periodic and aperiodic activity accounts for inter-trial variability in the perception of the McGurk illusion.

## Introduction

Perception is driven by incoming stimuli and brain's internal state (Van Dijk et al., 2008). The role of internal state in shaping perception has been investigated in both unisensory (Kayser et al., 2016; Krasich et al., 2022) and multisensory domains (Kaiser et al., 2019; Rohe et al., 2019). Evidences suggest that spontaneous neural (prestimulus) activity is related to variability in multisensory speech perception (Keil et al., 2012; Spadone et al., 2020). The significance of prestimulus activity in perception has been studied by examining the oscillatory properties including spectral power and phase synchrony in canonical frequency bands (Senkowski and Engel, 2024). However, studies suggest that traditional oscillatory activity is often confounded with aperiodic (scale-free) activity that complicates the computation and interpretation of spectral properties across frequency bands (Donoghue et al., 2020a). The aperiodic activity follows a 1/*f*-like distribution with exponentially decreasing power across increasing frequencies (Miller et al., 2009; He, 2014). It can further be parameterized with a slope (exponent) and intercept (offset) parameter (Donoghue et al., 2020b). These two parameters are known to reflect certain physiological information. Offset has been associated with underlying neuronal spiking (Manning et al., 2009) and exponent with excitation–inhibition (E/I) balance (Gao et al., 2017). Aperiodic components have also been linked to distinct cognitive states (Podvalny et al., 2015) that serve as neural makers for neuropathologies (Levin et al., 2020; Belova et al., 2021) and aging (Voytek et al., 2015; Thuwal et al., 2021). Although often considered distinct from oscillatory activity, some evidence suggests that both might originate from the same underlying neuronal population dynamics. Gireesh and Plenz (2008) demonstrated that neuronal avalanches in developing cortical networks organize as nested theta and beta/gamma oscillations. Similarly, Haegens et al. (2011) reported that alpha (8–14 Hz) oscillations are negatively correlated with neuronal spiking rates in the somatosensory cortex, underscoring their interdependence. Together, these findings suggest that oscillations and aperiodic activity, whether originating from shared or distinct mechanisms, represent complementary facets of brain dynamics whose balance might shape cognition.

Even within oscillatory (periodic) activity, parameters such as center frequency, aperiodic adjusted power, and bandwidth carry meaningful cognitive information. For instance, shifts in alpha center frequency are associated with increased cognitive demands (Haegens et al., 2014) and perceptual influences (Mierau et al., 2017). Although the role of bandwidth remains underexplored, studies suggest that together with center frequency, it provides insights into neural synchrony and circuit dynamics (Cole and Voytek, 2019). Lowet et al. (2022) suggest that variability in oscillatory parameters might reflect changes in neural computations across cognitive tasks. Therefore, examining changes in these parameters are crucial for understanding cognitive differences. However, in multisensory perception, it remains unclear how prestimulus periodic and aperiodic activity together influence perception in lower-level areas (occipital and temporal) and possibly higher-level areas (frontal and parietal). Therefore, this study aimed to identify the separate and interactive contributions of prestimulus periodic power and aperiodic components to perceptual integration.

Studies on multisensory speech perception have predominantly employed the McGurk effect, wherein participants report an illusory percept when presented with a mismatching AV stimulus (e.g., auditory */ba/* dubbed over visual */ga/* producing */da/*; McGurk and MacDonald, 1976). The likelihood of illusory perception varies across individuals and is not consistent across trials (Nath and Beauchamp, 2012; Kumar et al., 2020). This makes it a lucrative paradigm for investigating the role of prestimulus activity in multisensory perception. Keil et al. (2012) reported an increased beta power (14–30 Hz) in the left superior temporal gyrus (lSTG), precuneus, and right frontal cortex before perceiving the McGurk illusion. However, the difference in the spectral power were computed without separating the aperiodic activity from the periodic power and activity changes were observed for averaged trials, limiting insights into trial-by-trial perceptual variability.

Summing up a consensus emerges that there lacks a comprehensive understanding of how prestimulus spectral features from different brain regions influence trial-wise perceptual responses. To address this, we reanalyzed EEG data from Kumar et al. (2020), examining the differences in prestimulus periodic and aperiodic components between illusory and nonillusory McGurk trials, thereby capturing trial-wise variability. We used logistic mixed-effects models to interpret which spectral parameters, periodic (center frequency, aperiodic adjusted power, bandwidth) and aperiodic (offset, exponent), predicted the perceptual outcome across sensor regions. We also fitted logistic mixed-effects interaction models to understand the synergistic relationship between prestimulus aperiodic adjusted power and aperiodic exponent that affected subsequent illusory percept at single trial level. Overall, we showcase how prestimulus periodic power and aperiodic activity distinctly and together index the veracity and experience of multisensory illusory speech perception.

## Materials and Methods

### Participants

For this study, we have used previously recorded EEG and behavioral data described in Kumar et al. (2020), where 18 right-handed healthy participants (eight females) with a mean age of 24.9 (SD = 2.8 years) were recruited for the study. All participants had normal or corrected-to-normal vision and had normal hearing. A written informed consent was obtained from the participants under the experimental protocol approved by the Institutional Human Ethics Committee (IHEC).

### Stimulus design and trials

Each participant viewed 600 trials of videos of a male annotator (matched by the geographic region and demographic background of the participants) articulating the syllables */pa/*, */ta/*, and */ka/*. Participants were instructed to report their subjective perceptions of the audiovisual (AV) stimuli. The experiment included four types of AV stimuli: three congruent (audio syllables matching video articulation) syllables */pa/*, */ta/*, and */ka/* and one McGurk (audiovisual mismatch) syllable (auditory */pa/* with visual */ka/*), which created the illusion of syllable */ta/*. Participants responded to the four randomly presented stimuli by reporting whether they heard */pa/*, */ta/*, */ka/*, or “*something else*,” unaware of the incongruent McGurk trial. The experiment was carried out for five blocks, where each block consisted of 120 trials (30 trials of each AV stimuli presented at random). A total of 150 incongruent McGurk trials out of 600 trials were presented to each participant. Interstimulus intervals were pseudorandomly varied between 1,200 ms (milliseconds) snd 2,800 ms to minimize expectancy effects (Minor, 1970).

### EEG data acquisition and preprocessing

A Neuroscan EEG recording and acquisition system (SynAmps2, Compumedics) with 64 Ag/AgCl scalp electrodes molded on an elastic cap in a 10–20 montage was used to collect continuous EEG scans, where the reference electrode was located near to Cz. The sampling rate was 1,000 Hz, and the channel impedances were kept below 10 kΩ.

The continuous EEG data collected was reanalyzed in the current study. Before analysis, raw EEG data were preprocessed using EEGLAB toolbox (Delorme and Makeig, 2004) and custom MATLAB codes (version R2020a, https://www.mathworks.com/). A linear (zero-phase) noncausal finite impulse response (FIR) filter was applied at 0.1 and 80 Hz to the data, followed by a ninth-order two-pass Butterworth filter (notch filter) between 46 and 54 Hz to remove the line noise. Any bad channels present—defined by extreme amplitude fluctuations or high variance or flatlines—were removed by visual inspection and interpolated using spherical spline interpolation (Perrin et al., 1989). Mean number of channels interpolated was 0.17 (SD = 0.38). The data was then average rereferenced. Eyeblinks and muscle artifact components from the signal were identified and removed through a systematic independent component analysis (ICA) pipeline. First ICA was applied on the continuous EEG signals using the *runica* function in EEGLAB. Then, each ICA component was classified into different categories of signals such as the eye, muscle, brain, and others by using an automatic EEG independent component classifier called *ICLabel* (Pion-Tonachini et al., 2019). Finally, components labeled eye and muscle with a probability of 0.9 and above were flagged and then removed. Mean number of ICA components removed was 1.95 (SD = 2.1).

Epochs of 0.8 s (−0.8 to 0 s) before the McGurk stimulus onset (prestimulus) and 0.8 s post the onset (poststimulus) were extracted (Fig. 2*A*). The extracted epoch data were then sorted based on illusory */ta/* and nonillusory */pa/* responses to the McGurk stimuli. The sorted prestimulus and poststimulus epochs were then baseline corrected by removing the temporal mean of the EEG signal on an epoch-by-epoch basis. Finally, to remove response contamination from any other artefacts, epochs with amplitudes above and/or below ±100 µV were removed from all electrodes. The mean number of epochs in the prestimulus duration discarded after thresholding was 17.69 (SD = 24.8) and mean number of epochs discarded in the poststimulus duration after thresholding was 9.17 (SD = 19.06).

### Spectral analysis

We computed the spectral power at each electrode on a trial-by-trial basis using the multitaper fast Fourier transform (*mtfft*) method, for both the prestimulus and the poststimulus epoch data. Power spectra were computed to extract the distribution of signal power over different frequency bands [theta (4–7 Hz), alpha (8–12 Hz), beta (15–30 Hz), and gamma (31–45 Hz)] for the incongruent McGurk stimulus condition using the Chronux toolbox function “*mtspectrumc.m*” (Bokil et al., 2010) and customized MATLAB codes. The time bandwidth product and the number of tapers used were set to 3 and 5, respectively.

### Extracting the periodic and aperiodic components from the power spectral densities

To separate the background 1/*f* aperiodic component from its periodic counterpart, we used modified version of SpecParam v1.0.0 (previously called FOOOF or Fitting Oscillations and One Over *f*, https://github.com/fooof-tools/fooof) algorithm (Donoghue et al., 2020b). The algorithm takes the original power spectral densities (PSDs), extracts the aperiodic signal, and superimposes them on periodic components, referred to as “peaks.” These peaks are oscillations and are modeled individually as Gaussian functions. Each of these Gaussians has three parameters that are used to define a periodic oscillation: center frequency (CF) or peak frequency, power (PW) of the peak—which is the distance between the peak of the Gaussian and the aperiodic fit—and bandwidth (BW) as two standard deviations. The aperiodic component is defined by two parameters: exponent or negative slope (Freeman and Zhai, 2009) and offset, which is the *y*-intercept of the model fit across frequencies (Manning et al., 2009).

The periodic and the aperiodic components were extracted from the prestimulus and poststimulus power spectra of each electrode, trial-wise using the MATLAB implementation of SpecParam/FOOOF (version 1.0.0; Donoghue et al., 2020b). The following model fit settings were applied: peak width limits, 0.512 Hz; maximum number of peaks, infinite; minimum peak height, 0.0; peak threshold, 2.0 SDs; aperiodic mode, fixed; and evaluated frequency range, 1–45 Hz. The frequency range was chosen based on model fit results with different algorithm settings on a single participant data (see Extended Data Fig. 3-2 for more details). The aperiodic fit estimated by the model was computed in logarithmic space. So, to extract the periodic power, aperiodic fit was transformed back to linear space and subtracted from the original power spectrum (Gyurkovics et al., 2022). The topographical distributions of both periodic parameters (center frequency, peak power, and bandwidth) and aperiodic parameters (offset and exponent), as well as the mean periodic power across different frequency bands, were examined.

### Statistical analysis

#### At the group level

To examine group-level differences in spectral power (both prestimulus and poststimulus) between illusory (*/ta/*) and nonillusory (*/pa/*) trials across frequency bands, a repeated-measures analysis of variance (ANOVA) was performed using MATLAB's *ranova* function. The analysis employed a 2 (condition: */ta/*, */pa/*) × 4 (frequency: theta, alpha, beta, gamma) within-subject design. For each participant, mean power values were computed for each frequency bands and condition, and these were fit into the repeated-measures ANOVA model. One participant with 100% illusory */ta/* McGurk susceptibility was excluded from the group-level pairwise analysis. Sphericity was evaluated using Mauchly's test, and Greenhouse–Geisser corrections for *p* values (*p_GG_*) were applied when the assumption of sphericity was violated. Partial eta-squared (*η*^2^) was calculated to estimate effect sizes for main and interaction effects. Post hoc pairwise comparisons between condition within each frequency band were performed using Tukey’s HSD test for multiple-comparison corrections. To further dissociate condition effects on periodic power and aperiodic components, a similar repeated-measures ANOVA was conducted on parameterized spectral features. This analysis used a 2 (condition: */ta/*, */pa/*) × 6 (component: mean theta, alpha, beta, gamma power; aperiodic offset, aperiodic exponent) within-subjects design. The same procedures for sphericity assessment, correction, effect size estimation, and post hoc testing were applied as in the power spectral analysis.

#### At the sensor level

To assess differences between illusory */ta/* and nonillusory */pa/* conditions at the sensor level, we used a mass univariate linear mixed-effects regression (LMER) approach implemented in the *lme4* package (v1.1.32; Bates et al., 2015) in R (R Core Team, 2022). A separate model was fit at each sensor, with the spectral feature of interest—mean periodic power across frequency bands, aperiodic offset, or aperiodic exponent—modeled as a function of *Condition* (fixed effect), while including *Subject ID* as a random intercept to account for repeated measures and interindividual variability. The model formula was as follows:

Values∼Condition+(1|SubjectID),

where Values denotes the spectral feature of interest at each sensor and trial. Condition reflects the two response conditions—illusory */ta/* and nonillusory */pa/*. To control for multiple comparisons, we implemented permutation testing at the sensor level. For each sensor, the condition labels were randomly permuted within each subject (preserving the within-subject structure) to generate a null distribution of the *t* value estimated for the condition effect (Maris and Oostenveld, 2007; Visalli et al., 2024). This process was randomized for 1,000 iterations per sensor. The observed *t* value was compared with the null distribution to compute a two-tailed permutation *p* value for each sensor. To further control the false discovery rate (FDR) arising from multiple statistical tests across all sensors and spectral features, we applied the Benjamini–Hochberg procedure to jointly adjust the permutation *p* values (Benjamini and Hochberg, 1995). Sensors and features with FDR-corrected *p* values below the significance threshold (alpha = 0.05) were considered as significant, ensuring robust inference across the entire set of comparisons.

### Predicting response to the McGurk trials from prestimulus parameterized power

We were interested to estimate which prestimulus periodic parameters (CF, PW, BW) and aperiodic parameters (offset and exponent) predicted behavioral responses to the upcoming McGurk trials across different sensor regions (Table 1). To do this, we used several logistic mixed models to analyze both whole-brain predictors and sensor region-specific predictors that significantly explained the behavior. The mixed models were fitted using the “*glmer*” function from the “*lme4*” package v.1.1.32 (Bates et al., 2015) in R (R Core Team, 2022), with *p* values provided by “*lmerTest*” v.3.1.3 package (Kuznetsova et al., 2017). To assess topographical variations in these periodic and aperiodic parameters, and for better overall model fit, the entire EEG scalp was further divided into six distinct, nonoverlapping sensor regions: frontal (14 sensors), central (14 sensors), parietal (14 sensors), occipital (14 sensors), left temporal (5 sensors), and right temporal (5 sensors; Table 1). To control for multiple comparisons across all frequency models, *p* values of all the model predictors were adjusted using the Benjamini–Hochberg method (Benjamini and Hochberg, 1995), maintaining the FDR at *α* = 0.05. The two possible behavioral outcomes (i.e., illusion vs nonillusion /pa/) were chosen as the dependent variable. The model fitted parameters were estimated using bound optimization by quadratic approximation (BOBYQA) optimizer with a set maximum of 200,000 iterations (Powell, 2009). The periodic parameters (center frequency CF, aperiodic adjusted peak power PW, and bandwidth BW) and aperiodic parameters (offset, exponent) were chosen as fixed effects (or predictors) of the models. Since, we were interested to determine features that could individually predict the behavior, we assumed that the effect of each predictor on the dependent variable is independent of other predictors in the model. Moreover, to account for the interindividual variability, we used subject ID as the random effect. The fixed effects were mean-centered around zero (Hox et al., 2017). The general formula was defined as follows:

$$\begin{array}{rl} \log\;\left(\frac{P_{\mathrm{illusion}}}{1-P_{\mathrm{illusion}}}\right) & ∼\;\beta 0+(\beta 1.CF_{\mathrm{theta}}+\beta 2.PW_{\mathrm{theta}}+\beta 3.BW_{\mathrm{theta}})\\ & \;+(\beta 4.CF_{\mathrm{alpha}}+\beta 5.PW_{\mathrm{alpha}}+\beta 6.BW_{\mathrm{alpha}})\\ & \;+(\beta 7.CF_{\mathrm{beta}}+\beta 8.PW_{\mathrm{beta}}+\beta 9.BW_{\mathrm{beta}})\\ & \;+\;(\beta 10.CF_{\mathrm{gamma}}+\beta 11.PW_{\mathrm{gamma}}+\beta 12.BW_{\mathrm{gamma}})\\ & \;+\;(\beta 13.\mathrm{Offset}+\beta 14.\mathrm{Exponent})+(1|\mathrm{Subject}\;\mathrm{ID}), \end{array}$$

where *P*_illusion_ is the probability of perceiving the illusion, *β*_0_ is the intercept, and *β_n_* are the regression weights. The predictors included are the CF which is the center frequency, PW is the peak power, and BW is the bandwidth across different frequency bands (theta, 4–7 Hz; alpha, 8–12 Hz; beta, 15–30 Hz; and gamma, 31–45 Hz). These continuous variables define the periodic component of the power spectra. The aperiodic component was defined by offset and exponent. Prediction analyses were performed at the level of individual trial–region combinations. Each participant completed 150 McGurk trials, resulting in a potential total of 18 × 150 = 2,700 trials. Following EEG preprocessing and exclusion of trials with missing or incorrect behavioral responses (responses provided after the stimulus presentation), parameterized spectral features (center frequency, peak power, and bandwidth) were extracted from six anatomically defined sensor regions (frontal, central, parietal, occipital, left temporal, right temporal; Table 1). A region–trial pair was included in the analysis only if valid periodic parameters were identified by the SpecParam/FOOOF model in all four frequency bands (theta, alpha, beta, gamma). Region–trial pairs with missing values in any frequency band were excluded. This resulted in a total of 2,639 valid region–trial data points used in the logistic mixed-effects models. This stringent criterion ensured that all model predictors were based on reliably detected oscillatory activity (Donoghue et al., 2020b, 2021). Standardized parameters of the model were obtained by fitting the models on a standardized version (predictor values were mean-centered around zero) of the dataset, where the significance of each beta coefficient was tested against zero (i.e., *B_n_* = 0). The 95% confidence interval (CI) and *p* values were computed using a Wald *z*-distribution approximation (Demidenko, 2020).

**Table 1.** List of sensors categorized into respective sensor regions

Sensor regions	Electrodes
Frontal	F7, F5, F3, F1, Fz, F2, F4, F6, F8, FP1, FPz, FP2, AF3, AF4
Central	FC5, FC3, FC1, FCz, FC2, FC4, FC6, C5, C3, C1, Cz, C2, C4, C6
Parietal	P5, P3, P1, Pz, P2, P4, P6, CP5, CP3, CP1, CPz, CP2, CP4, CP6
Occipital	PO9, O1, Oz, O2, PO10, PO7, PO5, PO3, POz, PO4, PO6, PO8
Left temporal	FT7, T7, TP7, TP9, P7
Right temporal	FT8, T8, TP8, TP10, P8

We classified all the 64 EEG sensors into six sensor regions covering bilateral: frontal, central, parietal, occipital, and unilateral left temporal and right temporal.

Furthermore, to quantify the evidence of each predictor in the estimated logistic mixed models, we computed the Bayes factor (BF) of each independent predictor. We first conducted Bayesian analysis using the “*brms*” Stan package (version 2.21.0; Bürkner, 2017; Carpenter et al., 2017) in R. The Bayesian generalized (non)linear multivariate multilevel (or *brm*) model was fitted using weakly informative priors to avoid overfitting. Specifically, we used normal priors with a mean of 0 and a standard deviation of 3 for the intercept; followed by a normal prior with a mean of 0 and a standard deviation of 1 for fixed effects. Priors of the random effects were specified as half-Cauchy distributions with a location parameter of 0 and a scale parameter of 0.1. The model was estimated using Markov chain Monte Carlo (MCMC) sampling with four chains, each running for 2,000 iterations. To assess the strength of evidence for each predictor, we computed Bayes factors using the “*bayes_factor*” function within the “*brms*” package. Bayes factors were calculated by comparing *brm* models with and without each predictor of interest. Specifically, for each predictor, we compared the full model, which included all predictors, to a reduced model where the predictor of interest was removed. Following the guidelines by Jeffreys (1939) and Lee and Wagenmakers (2014), we interpreted Bayes factors as follows: a Bayes factor (BF) greater than 3 was considered moderate evidence in favor of the model with the predictor, a BF greater than 10 was considered strong evidence, and a BF greater than 100 was considered extreme evidence in favor of the model with the predictor. Conversely, BFs less than 1/3, 1/10, and 1/100 were interpreted as moderate, strong, and decisive evidence against the model with the predictor, respectively.

### Correlation analysis

The association between prestimulus mean aperiodic adjusted periodic power (across frequency bands) and aperiodic parameters (offset and exponent) was examined using Spearman rank correlation. The mean aperiodic adjusted periodic power was obtained by subtracting the aperiodic fit in linear space from the original untransformed spectrum (Gyurkovics et al., 2022). Correlations were computed for illusory */ta/* and nonillusory */pa/* trial conditions separately. To compute these associations, channel and trial-averaged aperiodic adjusted periodic power and aperiodic parameters were computed for each participant. Participants with zero trials in a given condition were excluded from that condition's correlation analysis.

### Logistic mixed-effects interaction models

**Figure 1.**
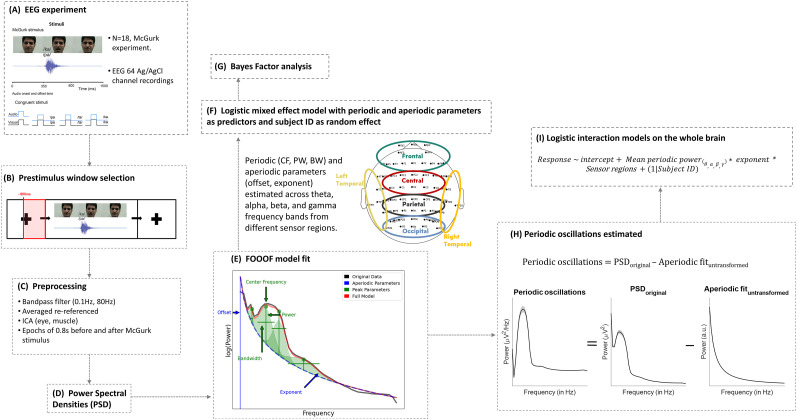
A schematic representation of the data processing and analyses pipeline. ***A***, Neurophysiological signals were recorded from 18 participants during the McGurk task using a 64-channel EEG system. ***B***, Prestimulus EEG data were extracted from a time window spanning −800 to 0 ms relative to stimulus onset. ***C***, The raw prestimulus EEG data was filtered, rereferenced, and subjected to independent component analysis (ICA) for artefact (eye and muscle) removal. ***D***, PSDs were estimated for all sensors and trials for all participants during illusory and nonillusory McGurk trial conditions. ***E***, Parameterization of the PSDs to estimate aperiodic and periodic activity using *SpecParam* (earlier called FOOOF) model. From the model, periodic parameters, center frequency, peak power, and bandwidth, and aperiodic parameters, offset and exponent, were estimated for all 64 sensors, across different frequency bands: theta (4–7 Hz), alpha (8–12 Hz), beta (15–30 Hz), and gamma (31–45 Hz). The parameters from all 64 EEG sensors were classified into six sensor regions: frontal, central, parietal, occipital, left temporal, and right temporal (see topoplot). ***F***, These periodic and aperiodic parameters were subsequently fitted as predictors in sensor region-wise logistic mixed-effects models to predict the behavioral response. To counter for intersubject variability, subject ID was put as random effect in the model. ***G***, Bayes factor analysis was performed as a post hoc test to validate the evidence of significant predictors estimated in the regression model. ***H***, Aperiodic adjusted power was estimated by subtracting the original PSD from aperiodic fit in linear space, a.u., arbitrary units. ***I***, Frequency-wise logistic mixed-effects interaction models were fitted with frequency power (aperiodic adjusted), aperiodic exponent, and sensor regions as predictors. Subject ID was chosen as the random effect.

The association between prestimulus aperiodic slope (or exponent) and aperiodic adjusted periodic power in predicting the response to upcoming stimulus was computed using logistic mixed-effects interaction models (Fisher, 1992). The model was applied across different frequency bands (theta, alpha, beta, and gamma). The frequency-wise models had the following general formula:

$$\begin{array}{rl} \log\;\left(\frac{P_{\mathrm{illusion}}}{1-P_{\mathrm{illusion}}}\right)\; & ∼\;\beta 0+(\beta 1.\mathrm{Mean}\;\mathrm{Power}\;*\;\beta 2.\mathrm{Exponent}\;*\;\beta 3.\mathrm{Sensor}\;\mathrm{Regions})\\ & \;+(1|\mathrm{Subject}\;\mathrm{ID}), \end{array}$$

where *P*_illusion_ is the probability of perceiving the illusion, *β*_0_ is the intercept, and *β_n_* are the regression weights. The model constituted of continuous predictors: exponent and mean aperiodic adjusted power in the frequency bands of interest (i.e., theta, alpha, beta, and gamma) and sensor regions as categorical predictor (frontal, central, parietal, occipital, left temporal, and right temporal). Subject ID was chosen as the random effect. Post hoc analysis using the Wald chi-square test compared the *p* values of interaction terms using R package *car* (v3.0.12): Anova (type “III”; Fox and Weisberg, 2019). To control for multiple comparisons, *p* values obtained from the post hoc tests were adjusted using Benjamini–Hochberg method across all sensor region models. For exploratory visualization of associations between interacting predictors, adjusted predictions were estimated for the response using R function *ggpredict* from package *ggeffects* (v1.2.3; Brooks et al., 2017). An 83% confidence interval (CI) threshold was assumed for visualizations that correspond to the 5% significance level with nonoverlapping estimates (MacGregor-Fors and Payton, 2013). For the schematic overview of the entire study, see Figure 1.

### Code accessibility

The raw EEG data used in this study were reanalyzed from a previous study published by Kumar et al. (2020). Therefore, access to the raw EEG data can be obtained by contacting the corresponding authors of the original study. The preprocessed EEG data and all the relevant codes used in this study are available in the provided Open Science Framework (OSF) link. The code was implemented on a Dell Precision 5820 Tower using a Windows 10 Pro for Workstations operating system. The code/software described in the paper is freely available online at https://osf.io/degzm. The code is available as Extended Data.

10.1523/ENEURO.0431-24.2025.d1 Data 1 MATLAB and R codes for implementing FOOOF to parameterize power spectra and for fitting logistic mixed-effects models, respectively. Download Data 1, ZIP file.

## Results

### Variability in illusory percept observed within and between participants

**Figure 2.**
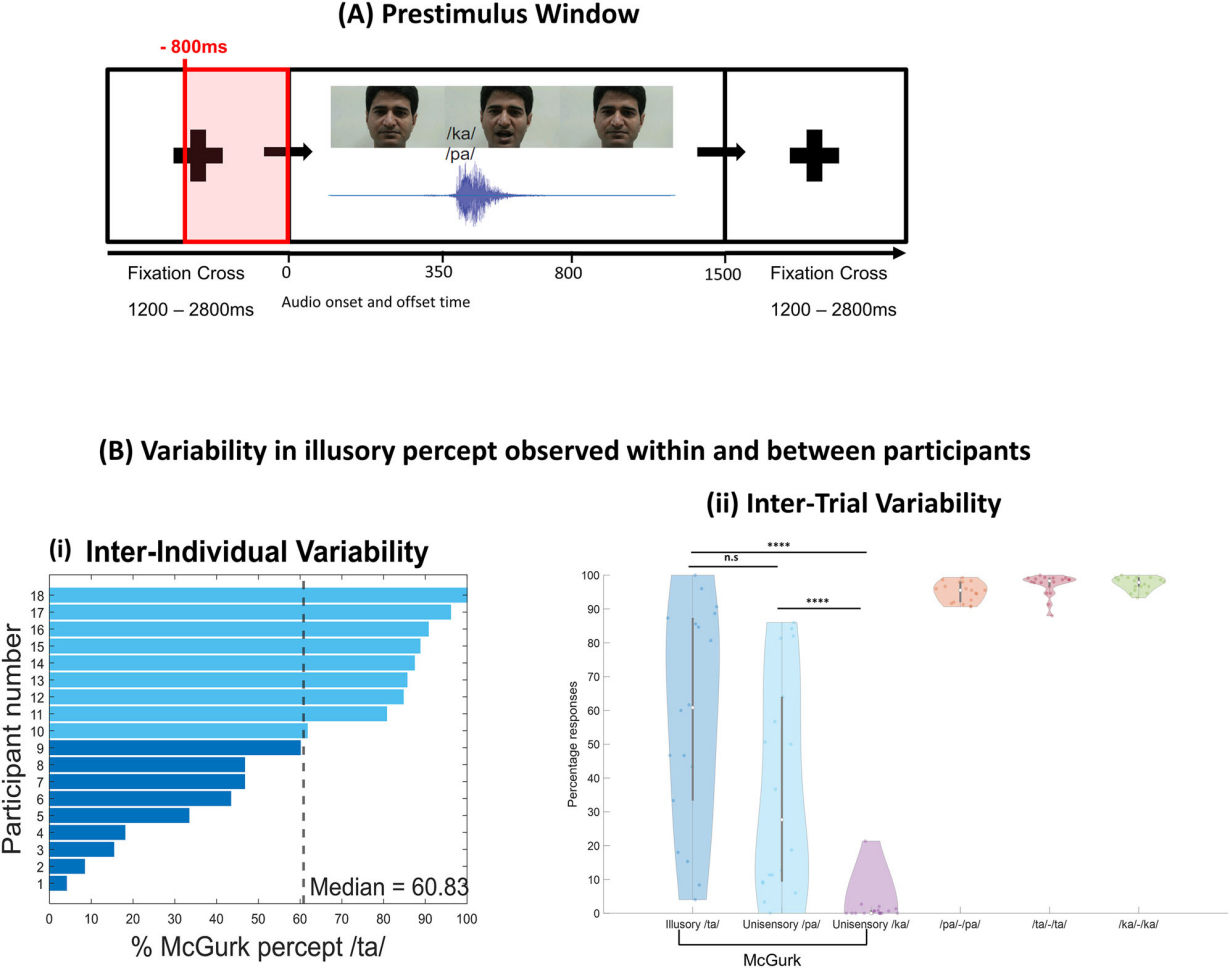
Prestimulus window relative to trial timing and behavioral results. ***A***, Example trial depicting three video frames from a video used in this experiment and intertrial interval (fixation cross) pseudorandomly varied between 1,200 and 2,800 ms. The red box indicates the 800 ms prestimulus epoch used in this study. ***B***, Behavioral results: ***i***, Bar graph representing interindividual variability–propensity of McGurk effect across 18 participants expressed as the percentage of */ta/* percept during the presentation of the McGurk stimulus. Dark blue represents participants below the median percentage of illusory response (or less prone), and light blue represents participants above the median percentage of illusory response (or more prone). ***ii***, Violin plot showing intertrial variability–percentage of */ta/* (illusory), unisensory */pa/* (auditory), and unisensory */ka/* (visual) percept during the presentation of McGurk stimulus and the congruent AV stimuli (*/pa/*, */ta/*, and */ka/*). The white dot in the center of each violin plot represents the median. Significance levels are denoted by asterisks: ****p ≤ 0.0001 and n.s. is not significant.

We observed a high degree of interindividual variability in McGurk susceptibility (Fig. 2*Bi*). McGurk susceptibility for each participant was quantified as the total percentage of illusory */ta/* responses to the McGurk stimulus. Across the 18 participants, the percentage of illusory responses ranged widely from 4 to 100% (indicating an illusory percept on every McGurk trial), with a median response of 60.83%. We also calculated the response tendency (intertrial variability), which is the relative proportion of illusory responses for all the McGurk trials across all 18 participants (Bechtold, 2016). For incongruent McGurk condition, illusion */ta/* was perceived in 58.38% (SD = 32.5) of trials, whereas unisensory */pa/* (auditory) was perceived in 37.39% (SD = 31.94) and unisensory */ka/* (visual) in 1.68% (SD = 4.97) of total trials presented across all participants. The remaining 2.55% of trials were either responded as “something else” (1.53% of total trials, SD = 2.85) or no response at all (1.02% of trials). To statistically compare response distributions, we performed nonparametric Friedman's test, which revealed a significant difference among the three response categories (*χ*^2^_(2)_ = 23.35, *p* < 0.001, Kendall's *W* = 0.65) to McGurk stimulus. Post hoc pairwise comparisons using the *multcompare* function in MATLAB with the Tukey’s HSD correction for multiple comparisons revealed no significance (*p* = 0.827) between the number of illusion and unisensory */pa/* (auditory) trials. However, significant differences between illusion */ta/* and unisensory */ka/* (visual; *p* < 0.001) and between unisensory */pa/* and unisensory */ka/* (*p* < 0.001) was observed (Fig. 2*Bii*). Because very few participants (8 out of 18 participants) reported the visual */ka/* response to the McGurk stimulus, we considered the auditory */pa/* response (17 out of 18 participants) as the nonillusory trial condition for subsequent analyses. This choice allowed for a more reliable within-subject comparison between illusory */ta/* and nonillusory */pa/* perception across trials. Response tendency was also estimated for the congruent trial conditions where participants correctly responded to congruent AV stimuli in 96.54% (SD = 2.66) of trials. We then proceeded with analyzing the electrophysiological data for both illusory */ta/* and nonillusory */pa/* McGurk trials.

### No group-level differences observed in periodic power and aperiodic activity between illusory and nonillusory McGurk trials

**Figure 3.**
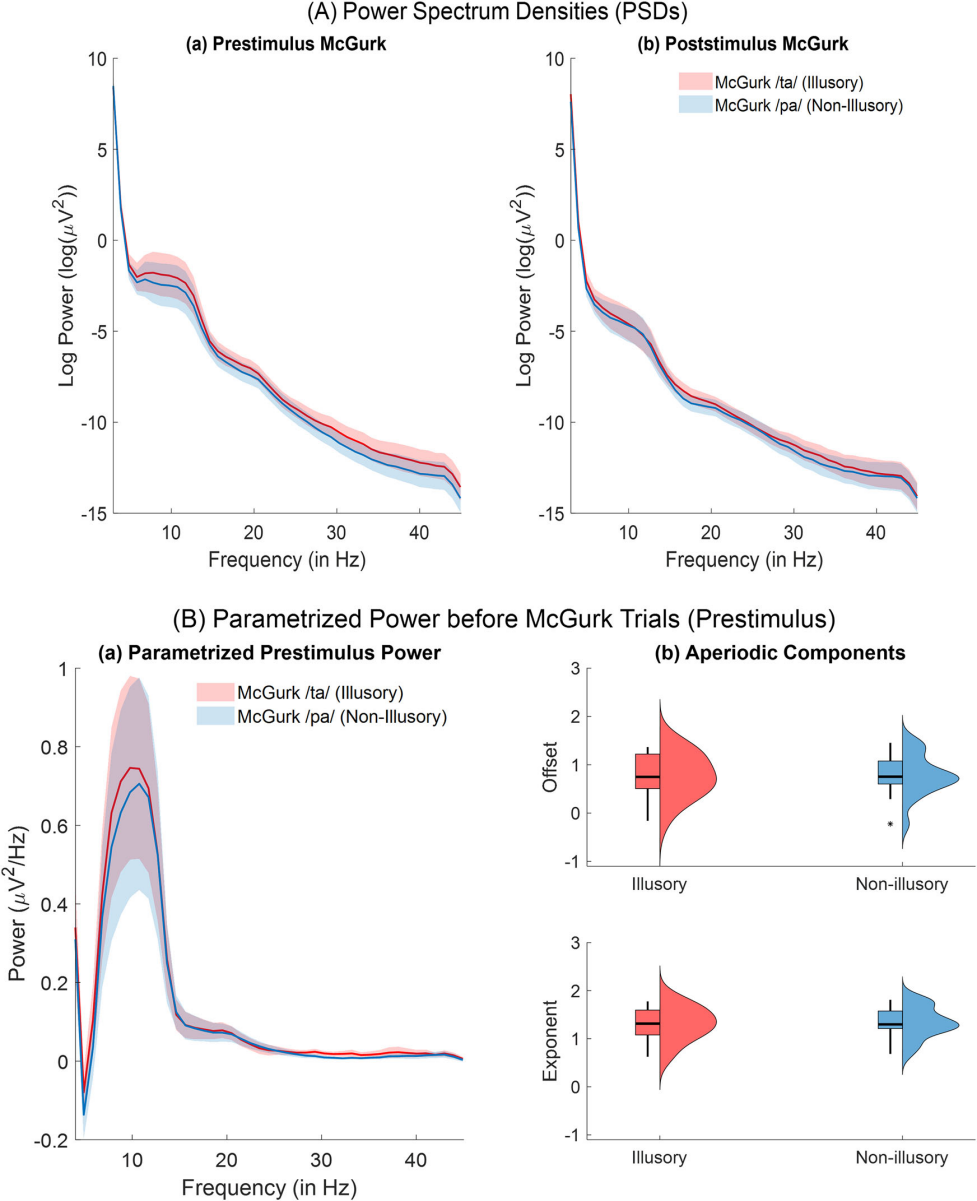
Grand-averaged PSDs and parameterized power distributions. ***A***, Group-averaged PSDs computed from subject-specific spectra, averaged over all sensors and trials within each participant. Mean power spectra are shown for illusory (red) and nonillusory (blue) McGurk trials, with shaded areas representing the standard error of the mean (SEM), separately for the (***a***) prestimulus and (***b***) poststimulus periods. ***B***, Prestimulus power distributions derived from parameterized spectra. Panel ***a*** shows grand-averaged periodic (oscillatory) power, averaged over all sensors and trials within each participant for illusory (red) and nonillusory (blue) conditions, with SEM shading. Panel ***b*** displays box-and-whisker plots overlaid with density distributions for the aperiodic parameters—offset (top) and exponent (bottom)—averaged over sensors and trials within each participant, grouped by perceptual condition (red, illusory; blue, nonillusory). All extended analysis of parametrized power distributions after perception of McGurk trials indicating interindividual (or group-level) variability is shown as Extended Data Figure 3-1 and two different model algorithms were fit on one participant prestimulus McGurk data is shown as Extended Data Figure 3-2.

A repeated-measures ANOVA with a 2 (condition: */ta/* and */pa/*) × 4 (frequency: theta, alpha, beta, gamma) within-subjects design was computed to analyze power spectrum differences between illusory and nonillusory response conditions. During prestimulus duration, the analysis revealed no significant interaction between condition and frequency (*F*_(3,48)_ = 0.239, *p_GG_* = 0.868, partial *η*^2^ = 0.015). However, post hoc pairwise comparison with Tukey’s HSD multiple correction revealed significantly higher beta power (mean difference = 0.34, 95% CI = [0.015, 0.662], *p* = 0.041, Cohen's *D* = 0.539) and higher gamma power (mean difference = 0.575, 95% CI = [0.229, 0.921], *p* = 0.003, Cohen's *D* = 0.854) before illusory perception (Fig. 3*Aa*). For poststimulus duration, the repeated-measures ANOVA revealed no significant interaction between condition and frequency (*F*_(3,48)_ = 0.529, *p_GG_* = 0.571, partial *η*^2^ = 0.032). Post hoc tests revealed no significant condition differences across frequency bands (Fig. 3*Ab*). Consistent with previous findings by Keil et al. (2012), we observed higher prestimulus beta band power associated with illusory perception; however, we did not observe a reduction in theta power following illusory perception.

10.1523/ENEURO.0431-24.2025.f3-1 Figure 3-1 Parametrized power distributions after perception of McGurk trials indicating inter-individual (or group-level) variability. (A) Poststimulus periodic power distributions for the illusory (red) versus non-illusory (blue) McGurk trials with SEM as shaded region. (B) Box and whisker plots with density distributions for comparisons between poststimulus aperiodic offset (above) and exponent (below), by response conditions (red – illusory, and blue – non-illusory). Download Figure 3-1, TIF file.

10.1523/ENEURO.0431-24.2025.f3-2 Figure 3-2 Tuning the SpecParam (or FOOOF) algorithm for better fit. To estimate the best model parameters, two different model algorithms were fit on one participant prestimulus McGurk data (*n* = 75). For model #1 (red), default fit parameters were chosen with frequency range of 1 – 45 Hz. For model #2 (blue), the frequency range chosen was 3 – 45 Hz. No significant difference between (A) aperiodic exponent estimated by the two model algorithms were observed (*Z* = -0.0113, *p* = .9910, Cohen’s D = 0.0837). (B) Visual inspection of mean absolute error (or MAE) indicated that both model algorithms fit the participant data well with majority of trials with MAE < 0.100. (C) Variance explained by model #1 (R^2^) was slightly higher (*M* = 0.98, *SD* = 0.04) as compared to model #2 (*M* = 0.97, *SD* = 0.02). Overall, model #1 demonstrated better goodness-of-fit (R^2^ and MAE), supporting our decision to use the 1–45 Hz frequency range in our study. Download Figure 3-2, TIF file.

We further examined the group-level differences in periodic power and aperiodic component estimated by parameterizing the power spectrum using SpecParam (FOOOF) model (Donoghue et al., 2020b). The periodic oscillations were computed after removing the aperiodic component in linear space from untransformed power spectrum from trials where peaks were detected after fitting the FOOOF model. A repeated-measures ANOVA with a 2 (condition: */ta/* and */pa/*) × 6 (component: mean theta, alpha, beta, gamma power; aperiodic offset, aperiodic exponent) within-subjects design was computed to analyse differences in periodic power and aperiodic component between illusory and nonillusory response conditions. We observed no significant interaction between condition and component for either the prestimulus (*F*_(5,80)_ = 0.343, *p_GG_* = 0.632, partial *η*^2^ = 0.021; Fig. 3*B*) or poststimulus duration (*F*_(5,80)_ = 1.548, *p_GG_* = 0.227, partial *η*^2^ = 0.090; Extended Data Fig. 3-1). Moreover, post hoc tests also revealed no significant pairwise condition difference for aperiodic components (offset and exponent) and periodic power across frequency bands. These findings emphasize the importance of separating aperiodic activity from periodic oscillations to accurately characterize the spectral changes associated with the McGurk illusory percept. Importantly, the absence of group-level effects prior to McGurk perception suggests that individual differences in susceptibility may obscure perception-related neural dynamics, making intertrial analyses a more sensitive approach to uncover the underlying mechanisms before McGurk perception. We then proceeded to capture the trial-wise variability at the sensor space.

### Spatial topographies of prestimulus periodic power and aperiodic component show distinct brain regions involved in illusory and nonillusory perception

**Figure 4.**
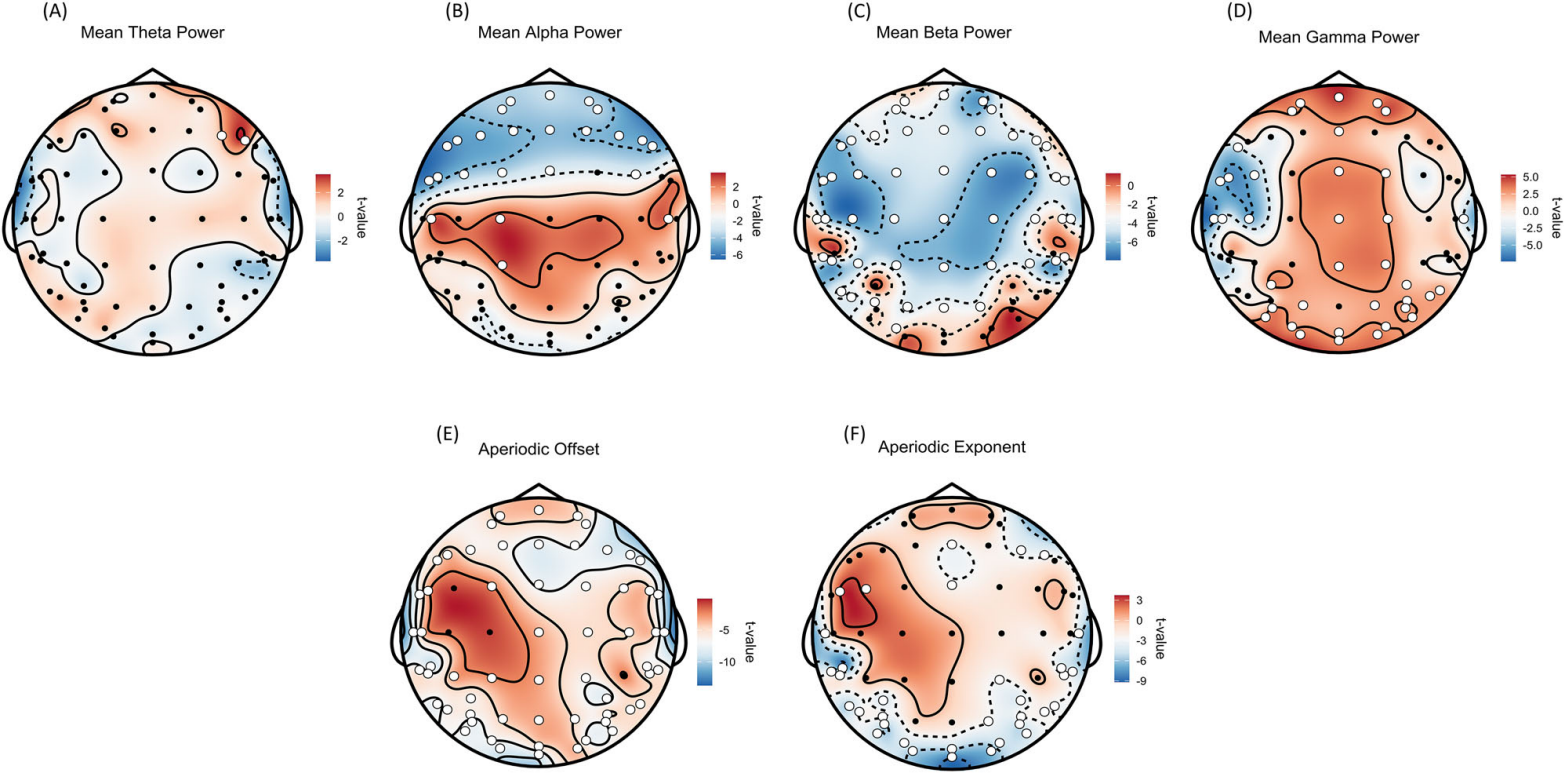
Topographic distribution of *t* values from linear mixed-effects models for periodic and aperiodic spectral components. Topoplots depicting *t* values from linear mixed-effects models comparing illusory (*/ta/*) and nonillusory (*/pa/*) McGurk trials for mean periodic power across (***A***) theta, (***B***) alpha, (***C***) beta, and (***D***) gamma frequency bands, as well as aperiodic components: (***E***) offset and (***F***) exponent. The color bar indicates the corresponding *t* values. White-marked sensors denote statistically significant differences between conditions. Periodic power was computed by subtracting the aperiodic fit from the original (untransformed) PSDs in linear space.

To investigate differences in prestimulus spectral activity preceding illusory */ta/* and nonillusory */pa/* perceptions, we compared trial-wise spatial topographies of periodic power (theta, alpha, beta, gamma) and aperiodic components (offset, exponent) using mass univariate linear mixed-effects models with permutation-based correction (see Materials and Methods section for details). Regression models revealed significant differences across a distributed set of scalp sensors after FDR correction (*p* < 0.05). Band-specific patterns revealed distinct topographical profiles. A higher mean theta power was observed over frontal F4 and F6 sensors before illusory perception (Fig. 4*A*). A lower mean alpha power was observed over frontal, frontal-central, and frontal-temporal sensors; whereas a higher alpha power over central sensors (C5, C1, C6) were observed before illusion (Fig. 4*B*). A lower beta power was observed across scalp before McGurk illusory perception (Fig. 4*C*). A higher gamma power over frontal, central, parietal, occipital, and right temporal sensors were observed; and a lower gamma power was observed over left temporal sensors before illusory perception (Fig. 4*D*). For the aperiodic offset, a higher *t* value was observed across scalp (Fig. 4*E*). And, finally lower aperiodic exponent was observed across temporal and occipital sensors and a higher exponent over FC3 and FC5 sensors was observed before illusory perception (Fig. 4*F*). Taken together, these spatial topographical distributions of mean periodic power and aperiodic component indicate distinct prestimulus brain states preceding the perception of an impending McGurk stimulus. These differences are evident not only in the unisensory perceptual regions like the occipital and temporal sensor regions but also in higher cognitive processing regions, such as the frontal, central, and parietal areas. So, to further understand how changes in these prestimulus spectral features relate to varying perception to McGurk stimulus, we further divided the whole scalp into six distinct sensor clusters covering bilateral frontal, central, parietal, occipital, and unilateral left temporal and right temporal sensors (see Table 1 for details). And then, fitted logistic mixed-effects models across the entire brain and different sensor regions.

### Prestimulus periodic and aperiodic parameters across different sensor regions can predict the response to the upcoming McGurk stimulus

**Figure 5.**
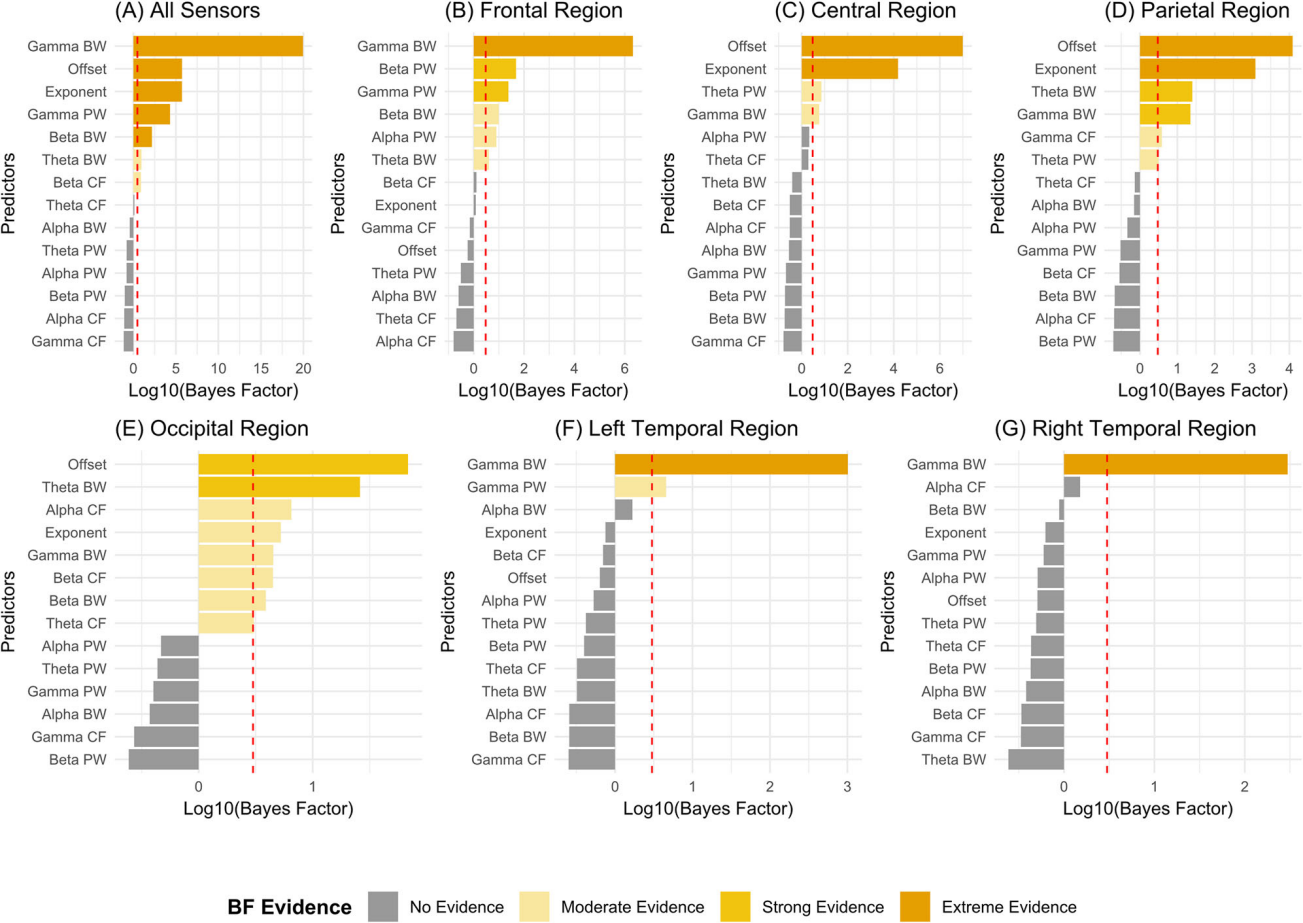
Bayes factor estimated for all the prestimulus periodic (CF, PW, BW) and aperiodic (offset, exponent) parameter predictors that were fitted to a logistic mixed-effects model to predict response to upcoming McGurk stimulus. The models were fit for (***A***) all sensor region, (***B***) frontal, (***C***) central, (***D***) parietal, (***E***) occipital, (***F***) left temporal, and (***G***) right temporal. The Bayes factor evidence scale was as follows: extreme evidence, BF > 100; strong evidence, 10 < BF < 100; moderate evidence, 3 < BF < 10; no evidence, BF < 3. For representation purposes, exact BF values have been log base 10 transformed. Summary tables of the logistic mixed-effects models fitted across the whole brain with periodic (CF, PW, BW) and aperiodic (offset, exponent) parameter as predictors is shown as Extended Data Figure 5-1. Summary tables of the logistic mixed-effects models fitted across the frontal, central, parietal, and occipital sensors are displayed as Extended Data Figures 5-2–5-5, and Extended Data Figures 5-6 and 5-7 show summary tables of the logistic mixed-effects models fitted across the left and right temporal sensors, respectively.

We fitted region-specific logistic mixed-effects models to predict illusory responses to incongruent McGurk stimuli. The models incorporated periodic parameters—center frequency (CF), aperiodic adjusted power at identified peaks (PW), and bandwidth (BW) across the theta, alpha, beta, and gamma frequency bands—as well as aperiodic parameters, including offset and exponent, as continuous predictors. Subject ID was included as a random effect (see Materials and Methods section for design of the model). As a measure of post hoc analysis, Bayes factor was estimated for all predictors to assess the evidence of significant predictors. At whole brain level, extreme evidence (BF > 100) of beta BW, gamma BW, gamma PW, offset, and exponent predictors was observed. For theta BW and beta CF, however, moderate evidence (3 < BF < 10) was predicted (Fig. 5*A*). Across different sensor regions, we found evidences for certain predictors that significantly predicted the illusory response to the impending McGurk stimulus. Aperiodic offset and exponent parameters from central and parietal sensors showed extreme evidence (BF > 100) for predicting the illusory response to the upcoming McGurk stimulus. Aperiodic offset from occipital sensors showed strong evidence (10 < BF < 100) and occipital exponent showed moderate evidence (3 < BF < 10) in predicting illusory responses. The influence of periodic parameter predictors, before illusory perception, proved to be more complex than initially expected. In the frontal region, extreme evidence (BF > 100) was found for gamma BW, with strong evidence (10 < BF < 100) observed for beta PW and gamma PW and moderate evidence (3 < BF < 10) for beta BW, alpha PW, and theta BW (Fig. 5*B*). In the central sensor region, moderate evidence was observed for theta PW and gamma BW (Fig. 5*C*). In the parietal region, strong evidence was found for theta BW and gamma BW and moderate evidence for gamma CF and theta PW (Fig. 5*D*). In the posterior occipital region, theta BW showed strong evidence for predicting the response to McGurk trials, while alpha CF, exponent, beta CF, gamma BW, beta BW, and theta CF showed moderate evidence (Fig. 5*E*). Finally, in the temporal region, extreme evidence was found for gamma BW, along with moderate evidence for gamma PW in the left temporal region (Fig. 5*F*,*G*). For more details on model output summaries and estimated Bayes factor values for each predictor, refer to Extended Data Figures 5-1–5-7. Overall, the results of logistic mixed-effects models show that different components of PSDs from different sensor regions predict the response to McGurk trials. Whether these parameters contribute to the illusory effect independently or dependently is what we looked into with correlation analysis.

10.1523/ENEURO.0431-24.2025.f5-1 Figure 5-1 Summary tables of the logistic mixed-effect models fitted across the whole brain with periodic (CF, PW, BW) and aperiodic (offset, exponent) parameter as predictors. Predictors that significantly predicted the response are in bold. Download Figure 5-1, TIF file.

10.1523/ENEURO.0431-24.2025.f5-2 Figure 5-2 Summary tables of the logistic mixed-effect models fitted across the frontal sensors. Predictors that significantly predicted the response are in bold. Download Figure 5-2, TIF file.

10.1523/ENEURO.0431-24.2025.f5-3 Figure 5-3 Summary tables of the logistic mixed-effect models fitted across the central sensors. Predictors that significantly predicted the response are in bold. Download Figure 5-3, TIF file.

10.1523/ENEURO.0431-24.2025.f5-4 Figure 5-4 Summary tables of the logistic mixed-effect models fitted across the parietal sensors. Predictors that significantly predicted the response are in bold. Download Figure 5-4, TIF file.

10.1523/ENEURO.0431-24.2025.f5-5 Figure 5-5 Summary tables of the logistic mixed-effect models fitted across the occipital sensors. Predictors that significantly predicted the response are in bold. Download Figure 5-5, TIF file.

**Figure 6.**
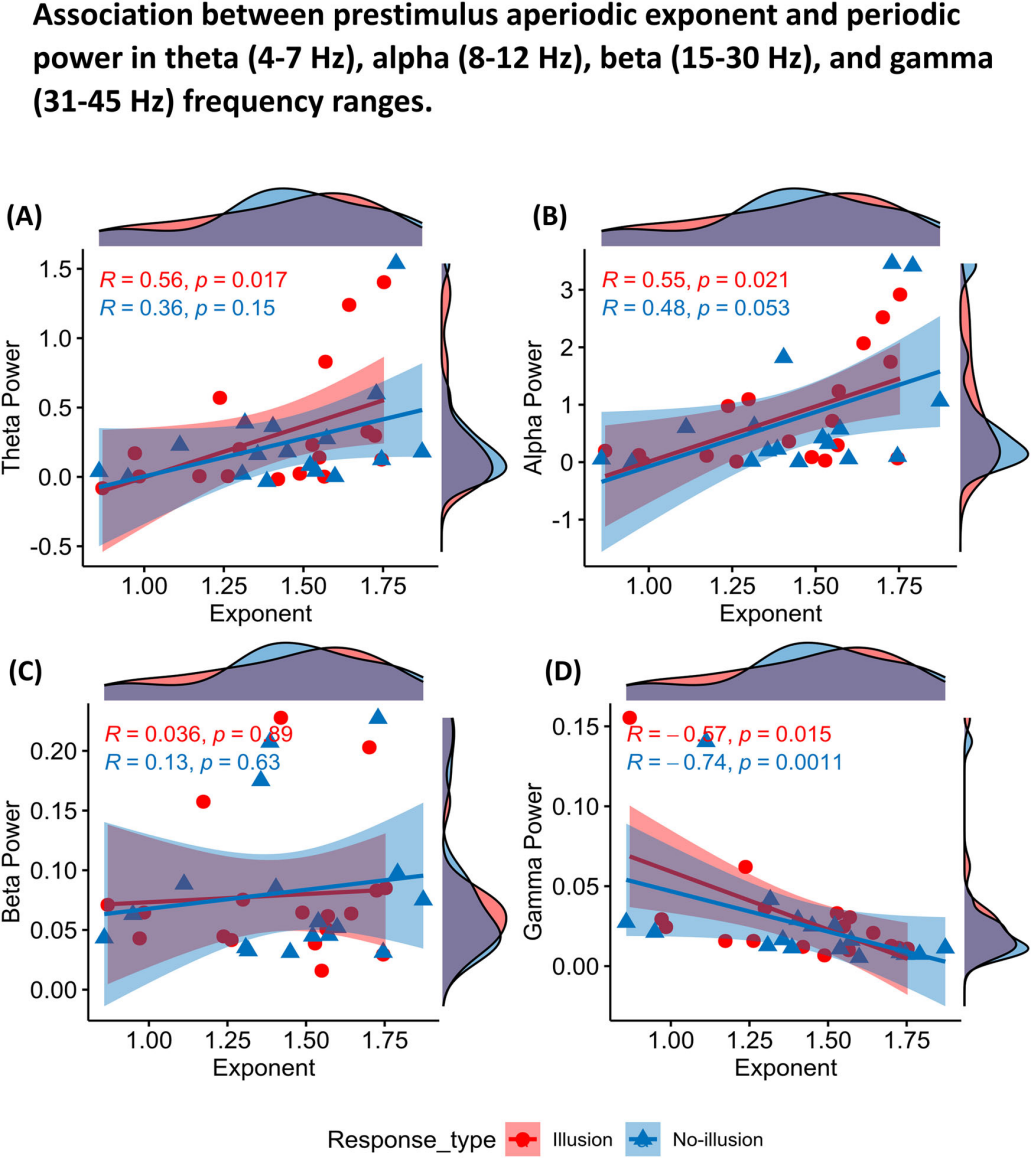
Association between aperiodic exponent and mean aperiodic adjusted power in the (***A***) theta, (***B***) alpha, (***C***) beta, and (***D***) gamma frequency bands before perception of illusory (red) and nonillusory (blue) McGurk trials averaged across all channels and trials for each participant. The aperiodic slope is represented on the *x*-axis and aperiodic adjusted power on the *y*-axis. The density of observations for each association plot is indicated on the margins for illusory (red) and nonillusory (blue) trial conditions. One participant contributed zero nonillusory trials, so the nonillusory condition includes *N* = 17. All the extended analysis of association between aperiodic offset and mean periodic power, in the (***A***) theta, (***B***) alpha, (***C***) beta, and (***D***) gamma frequency bands before perception of illusory (red) and nonillusory (blue) McGurk trials averaged across all trials and channels for each participant is shown as Extended Data Figure 6-1.

10.1523/ENEURO.0431-24.2025.f5-6 Figure 5-6 Summary tables of the logistic mixed-effect models fitted across the left temporal sensors. Predictors that significantly predicted the response are in bold. Download Figure 5-6, TIF file.

**Figure 7.**
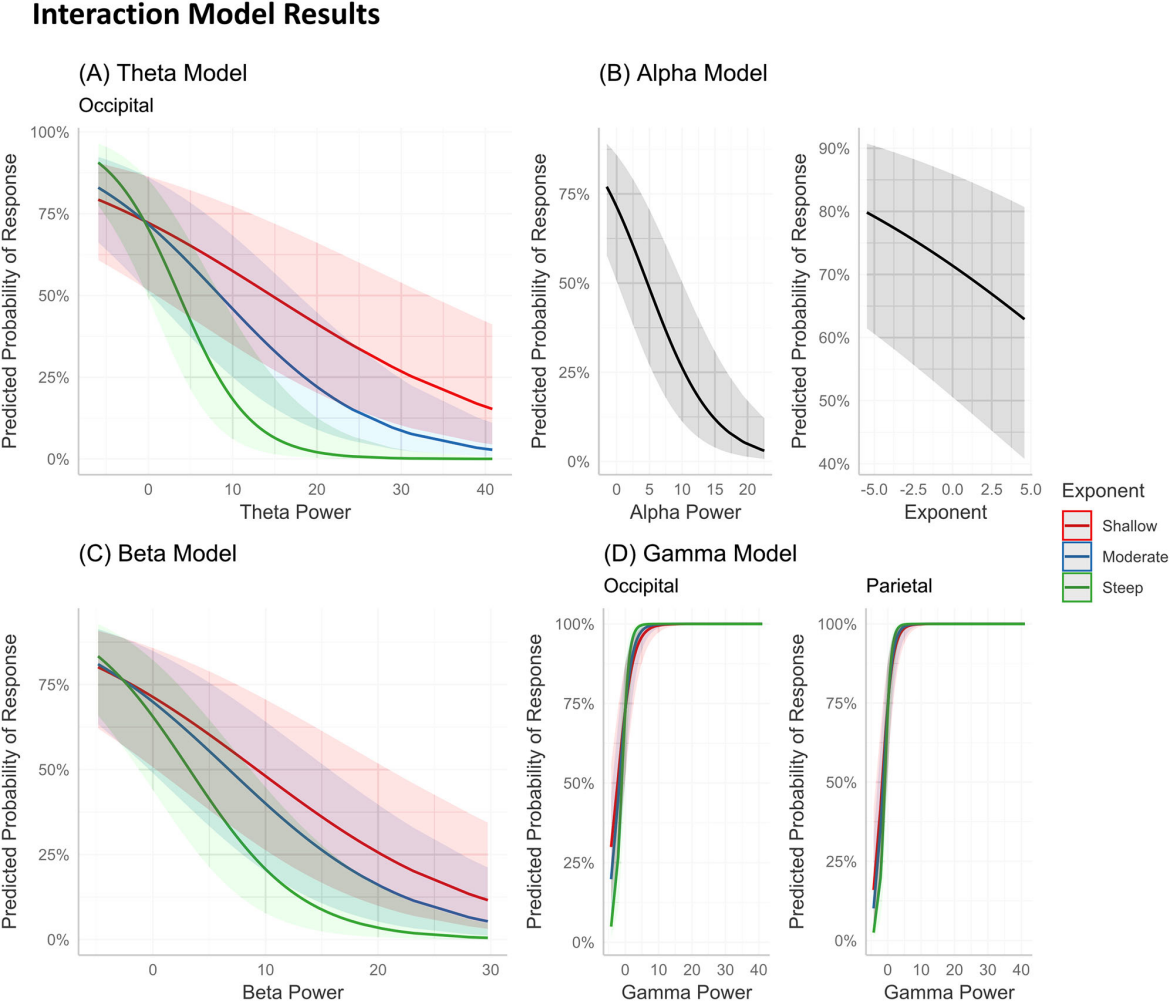
Visualization of the relationship between prestimulus aperiodic exponent and aperiodic adjusted mean power in predicting McGurk illusory response at (***A***) occipital theta, (***B***) alpha, (***C***) beta, and (***D***) occipital and parietal gamma band power. For ***A***, ***C***, and ***D*** frequency band models, *x*-axis represents the mean power (aperiodic adjusted) in the frequency range (higher values indicate higher power) and *y*-axis represents the probability (in percentage) of perceiving the illusion. Note that the distinction of aperiodic exponent into shallow, moderate, and steeper facets are for visualization purposes only and the aperiodic slopes were entered as continuous predictors in all the models. Also, note that ***B*** illustrates effect of individual predictors: alpha power (left; *x*-axis, higher values indicate higher power) and exponent (right; *x*-axis, absolute higher values indicate steeper slope) for prediction to response (*y*-axis). The shaded region indicates 83% CI.

10.1523/ENEURO.0431-24.2025.f5-7 Figure 5-7 Summary tables of the logistic mixed-effect models fitted across the right temporal sensors. Predictors that significantly predicted the response are in bold. Download Figure 5-7, TIF file.

### Association between mean periodic power and aperiodic parameters before perceiving the McGurk stimulus

By fitting logistic mixed-effects models, we observed that periodic and aperiodic activity from different regions could distinctly predict the response to the upcoming incongruent McGurk stimulus. We further examined the relationship between prestimulus mean periodic power at different frequency bands (theta, alpha, beta, and gamma) and aperiodic activity (offset and exponent) using Spearman rank correlation separately for illusory */ta/* and nonillusory */pa/* trial conditions. We found significant positive correlation for illusory conditions between mean theta power and exponent (*r*_(18)_ = 0.56, *p* = 0.017; Fig. 6*A*). A positive correlation was observed between mean alpha power and exponent for illusory (*r*_(18)_ = 0.55, *p* = 0.021) conditions (Fig. 6*B*). No significant association between mean beta power was observed for both illusory (*r*_(18)_ = 0.036, *p* = 0.89) and nonillusory (*r*_(17)_ = 0.13, *p* = 0.63) conditions (Fig. 6*C*). And, contrastingly, a negative correlation was observed between mean gamma power and exponent for both illusory (*r*_(18)_ = −0.57, *p* = 0.015) and nonillusory (*r*_(17)_ = −0.74, *p* = 0.0011) conditions (Fig. 6*D*).

10.1523/ENEURO.0431-24.2025.f6-1 Figure 6-1 Association between aperiodic offset and mean periodic power, in the (A) Theta, (B) Alpha, (C) Beta, and (D) Gamma frequency bands before perception of illusory (red) and non-illusory (blue) McGurk trials averaged across all trials and channels for each participant. The aperiodic offset is represented on the x-axis and oscillatory power on the y-axis. The density of observations for each association plot is indicated on the margins for illusory (red) and non-illusory (blue) trial parameters. Download Figure 6-1, TIF file.

For associations between mean power and offset for both response conditions, significant positive correlation was observed between mean theta power and offset (illusory: *r*_(18)_ = 0.67, *p* = 0.0031; nonillusory: *r*_(17)_ = 0.68, *p* = 0.0037) and a positive correlation between mean alpha power and offset (illusory: *r*_(18)_ = 0.73, *p* = 0.00086; nonillusory: *r*_(17)_ = 0.73, *p* = 0.0012). No significant association between mean beta power and offset (illusory: *r*_(18)_ = 0.17, *p* = 0.51; nonillusory: *r*_(17)_ = 0.16, *p* = 0.54) as well as mean gamma power and offset for both conditions were observed (illusory: *r*_(18)_ = 0.19, *p* = 0.45; nonillusory: *r*_(17)_ = −0.3, *p* = 0.25; Extended Data Fig. 6-1). These results suggest that narrowband frequency periodic power and aperiodic parameters especially slope (or exponent) may influence each other in shaping the perception of the impending McGurk stimulus. We further validated this association in predicting illusion by fitting a logistic mixed-effects interaction model across different frequency bands.

### Interactions between aperiodic parameters and mean periodic power predicts perception to McGurk stimulus

Given the association between prestimulus mean periodic power and aperiodic activity before perceiving the McGurk stimulus, we now examined whether the aperiodic exponent interact with power in the prestimulus duration to influence behavioral perception. We used logistic mixed-effects interaction models for each frequency band of interest (theta, alpha, beta, and gamma; see Materials and Methods section for more details on the model design) and found significant interactions between mean power and aperiodic activity (see Table 2 for ANOVA results for all interaction models). Significant interactions mean that the values of one independent predictor influence another's overall performance in prediction (Fisher, 1992). In case of the theta interaction model, we found a significant three-way interaction between theta power × exponent × sensor regions [*χ*^2^_(5)_ = 18.822, *p* (adjusted) = 0.0066]. This three-way interaction was most pronounced in the occipital sensor region (Log Odds = −0.082, 95% CI = [−0.16, −0.001], *p* = 0.038). The model revealed that when the exponent was steeper and as theta power lowered, the probability of illusory percept was higher (Fig. 7*A*). For the alpha interaction model, no significant two-way interaction [mean alpha power × exponent, *χ*^2^_(1)_ = 0.3849, *p* (adjusted) = 0.6795] or three-way interaction [mean alpha power × exponent × sensor regions, *χ*^2^_(5)_ = 9.951, *p* (adjusted) = 0.1752] was observed. However, both mean alpha power and the exponent were individually significant predictors [mean alpha power: *χ*^2^_(1)_ = 25.9706, *p* (adjusted) = <0.0001; exponent: *χ*^2^_(1)_ = 15.3560, *p* (adjusted) = 0.0005]. Figure 7*B* (left) illustrates that the probability of an illusory percept increases as alpha power decreases, while Figure 7*B* (right) shows that a steeper exponent is associated with a higher probability of an illusory percept. While the interaction was not statistically significant, this trend suggests a potential relationship between the predictors. For beta interaction model, there was a significant two-way interaction between beta power × exponent [*χ*^2^_(1)_ = 9.205, *p* (adjusted) = 0.0070]. As depicted in Figure 7*C*, when the exponent was the steepest and as beta power lowered, the likelihood of perceiving the illusion increased. Finally, for gamma interaction model, a significant three-way interaction between gamma power × exponent × sensor regions [*χ*^2^_(5)_ = 21.586, *p* (adjusted) = 0.0025] was observed. The effect was most pronounced in the occipital (Log Odds = 0.19, 95% CI = [0.06, 0.32], *p* = 0.0036) and parietal (Log Odds = 0.17, 95% CI = [0.05, 0.29], *p* = 0.0041) sensor regions, where when the exponent was steeper and as the gamma power increased, the probability of illusory percept was higher (Fig. 7*D*). Overall, these results suggest that when there is a steeper exponent, decreased theta and beta power were associated with better predicting the illusory response. Contrastingly, a higher gamma power associated with steeper exponent had a higher probability of perceiving the McGurk illusion.

**Table 2.** Fixed-effects ANOVA (type III Wald chi-square tests) results for logistic mixed interaction effects in the (A) theta, (B) alpha, (C) beta, and (D) gamma frequency bands

Fixed-effects ANOVA results for interaction models				
Predictors	Response			
	Chisq	DF	Pr (>Chisq)	*p* (adjusted)
(A) Theta model				
(Intercept)	2.1002	1	0.1473	0.2695
Theta power	**4.2466**	**1**	**0.0393**	0.0968
Exponent	**22.3972**	**1**	**<0.0001**	<0.0001***
Sensor regions	2.1016	5	0.8349	0.8618
Theta power × exponent	0.3536	1	0.5521	0.6795
Theta power × sensor regions	2.3918	5	0.7927	0.8455
Exponent × sensor regions	5.4656	5	0.3617	0.5512
Theta power × exponent × sensor regions	**18.8219**	**5**	**0.0021**	0.0066**
(B) Alpha model				
(Intercept)	1.9742	1	0.160	0.2695
Alpha power	**25.9706**	**1**	**<0.0001**	<0.0001***
Exponent	**15.3560**	**1**	**<0.0001**	0.0005***
Sensor regions	5.6547	5	0.3413	0.5461
Alpha power × exponent	0.3849	1	0.5350	0.6795
Alpha power × sensor regions	**19.9376**	**5**	**0.0013**	0.0046**
Exponent × sensor regions	5.0297	5	0.4122	0.5736
Alpha power × exponent × sensor regions	9.9510	5	0.077	0.1752
(C) Beta model				
(Intercept)	2.1394	1	0.1436	0.2695
Beta power	**12.9303**	**1**	**0.0003**	0.0015**
Exponent	**26.6636**	**1**	**<0.0001**	<0.0001***
Sensor regions	1.5859	5	0.9029	0.9030
Beta power × exponent	**9.2046**	**1**	**0.0024**	0.0070**
Beta power × sensor regions	2.8561	5	0.7221	0.8253
Exponent × sensor regions	5.2032	5	0.3916	0.5696
Beta power × exponent × sensor regions	3.5235	5	0.6198	0.7346
(D) Gamma model				
(Intercept)	1.9802	1	0.1594	0.2695
Gamma power	0.4862	1	0.4856	0.6475
Exponent	**18.3772**	**1**	**<0.0001**	0.0001***
Sensor regions	**17.0688**	**5**	**0.0044**	0.0116*
Gamma power × exponent	0.0775	1	0.7807	0.8455
Gamma power × sensor regions	**34.9677**	**5**	**<0.0001**	<0.0001***
Exponent × sensor regions	8.8578	5	0.1149	0.2451
Gamma power × exponent × sensor regions	**21.5860**	**5**	**0.00063**	0.0025**

Each model included mean periodic power and aperiodic exponent as continuous predictors. Significant predictor interactions are highlighted in bold. *p* values for all fixed effects were calculated using White-corrected covariance matrices (White, 1980). *p* values were adjusted for multiple-comparison corrections using Benjamini–Hochberg method. Significance levels are denoted by asterisks: *p ≤ 0.05, **p ≤ 0.01, ***p ≤ 0.001, and ****p ≤ 0.0001.

## Discussion

This study aimed to elucidate the role of prestimulus aperiodic component along with putative oscillations (or periodic power) in shaping perceptual response to upcoming McGurk stimulus. Accordingly, spectral features of EEG data obtained during the prestimulus duration were put in a statistical model where the dependent variable was the perceptual categorization of response to the McGurk stimulus. This analysis design allowed us to test the much broader hypothesis of whether the brain's internal states, further divided into periodic and aperiodic activity, are good predictors of individual perceptual experience. The prestimulus PSDs were parameterized to extract periodic and aperiodic components of the EEG signal at single trial level. We estimated the interindividual differences in the periodic power and aperiodic component in the context of McGurk effect. Subsequently, using mass univariate linear mixed-effects models with permutation-based correction, we investigated sensor region-wise differences in power and aperiodic parameters between the illusory */ta/* and nonillusory */pa/* conditions, independent of between-subject variability. Finally, using logistic mixed effect models, we evaluated whether the spectral markers of neural activity in the prestimulus period can predict the response to the upcoming McGurk trial, independent of interindividual variability. Four key findings emerged from this study: (1) no significant differences in power across frequency bands were observed across participants after adjusting for the aperiodic component prior to McGurk trials; (2) lower aperiodic exponent and offset from central, parietal, and occipital regions predict response to following McGurk stimulus on a single trial level; (3) periodic parameters [center peak frequency (CF), peak power (PW), and bandwidth (BW)] from specific sensor regions can also predict response to subsequent McGurk perception on a trial-by-trial basis; and (4) the steeper aperiodic exponent associated with lower occipital theta, global beta, and higher posterior gamma power shape the perception of the upcoming McGurk stimulus. These findings suggest that prestimulus periodic and aperiodic components reflect distinct brain states, and both individually and collectively influence the perception of upcoming AV stimuli at a single trial level of variability.

### Prestimulus periodic activity across different frequency bands leads to the perception of McGurk illusion

Periodic or rhythmic neural oscillations are known to encode different cognitive and behavioral states (Engel et al., 2001; Fries, 2005; Cole and Voytek, 2019). Keil et al. (2012) previously reported that prestimulus beta power correlated with the perception of the McGurk illusion. However, when we separated aperiodic broadband activity from periodic power using the FOOOF model, our repeated-measures ANOVA did not detect significant beta power differences between illusory and nonillusory conditions at the interindividual level (Fig. 3*A*). This suggests that the previously reported beta effects may have been influenced by underlying aperiodic activity, highlighting the importance of disentangling aperiodic and periodic components when interpreting power spectral differences related to McGurk perception. In contrast, our trial-wise sensor-level analysis revealed significantly higher prestimulus theta power over frontal sensors prior to illusion perception (Fig. 4*A*). Previous studies have demonstrated that increased theta power in frontal electrodes are associated with conflict processing, which reflects enhanced cognitive control following the perception of incongruent audiovisual stimuli (Hanslmayr et al., 2008; Cavanagh and Frank, 2014; Cohen, 2014; Ergen et al., 2014; Morís-Fernandez et al., 2018). In contrast, studies on McGurk effect perception suggest that reduced theta power after exposure to the McGurk stimulus primarily represents audiovisual integration (Keil et al., 2012; Lindborg et al., 2019). We propose that the elevated frontal periodic theta power observed in the prestimulus state before the illusory perception is linked to enhanced cognitive control or a shift in sensory precision (Cavanagh and Frank, 2014), likely directed toward the visual regions, which may contribute to the illusion. This hypothesis is supported by our logistic mixed-effects models (Fig. 5), which shows that in addition to increased frontal and central periodic theta power, elevated parietal and occipital periodic theta power predicts the illusory perception. However, a key caveat to consider with narrowband theta peaks is that the peak differences observed might stem from nonoscillatory spectral features (Donoghue et al., 2021) or shifts in neighboring bands (changes in alpha peak frequency or bandwidth; Seymour et al., 2022). Although we enforced stringent inclusion criteria so that only trials with valid periodic parameters in all four canonical bands were modeled, we cannot entirely rule out subtle cross-band interactions or transient nonoscillatory events contributing to our results. Future work that combines spectral parameterization with time-domain burst detection (Wen and Liu, 2016; Seymour et al., 2022; Wilson et al., 2022) or phase-based analyses (like cycle-by-cycle methods; Cole and Voytek, 2019) will help to further disambiguate true oscillations from spectral leakage or aperiodic fluctuations.

Moreover, lower prestimulus alpha band power has been associated with higher cortical excitability (Romei et al., 2008, 2010; Mathewson et al., 2009, 2011; Samaha et al., 2017). This means that at regions with high excitability (lower alpha power), the threshold of activation of the underlying neural population is lowered. Thus, improving the neural processing of sensory input leading to better perception, which in case of incongruent McGurk stimulus, sometimes may lead to illusory percept (Van Dijk et al., 2008; Jensen et al., 2012; Lange et al., 2014). In our study, we observed lower prestimulus periodic alpha power in the frontal and central sensor regions prior to illusory perception (Fig. 4*B*). This might reflect increased excitability or attentional engagement of higher-order networks that might be critical for multisensory integration in McGurk illusion (Foxe and Snyder, 2011; Haegens et al., 2012; Sadaghiani and Kleinschmidt, 2016). Fluctuations in frontal and central alpha power have also been linked to top-down modulation of attention and preparatory states that influence perceptual outcomes (Jones et al., 2010; Klimesch, 2012; Samaha et al., 2015). Notably, while we did not find significant occipital alpha power differences at the sensor level, our analysis of peak alpha frequency (PAF) in the occipital region (Fig. 5*E*) revealed that lower PAF predicted illusory percepts that is consistent with previous findings that occipital PAF modulates audiovisual integration and susceptibility to multisensory illusions (Cecere et al., 2015; Samaha and Postle, 2015; Keil and Senkowski, 2017; Venskus and Hughes, 2021). Taken together, these results suggest that lower prestimulus periodic alpha power in frontal and central regions, along with lower occipital PAF, may jointly facilitate the attentional and integrative processes that bias perception toward the McGurk illusion. This aligns with the view that alpha oscillations reflect both local excitability and large-scale network dynamics that shape multisensory perceptual experiences (Foxe and Snyder, 2011; Sadaghiani and Kleinschmidt, 2016).

Furthermore, we observed a significantly lower periodic beta power before illusory percept across all sensor regions (Table 1, Fig. 4*C*); however, our model could only predict response to illusion perception from periodic beta power over frontal and occipital electrodes (Fig. 5*B*,*E*). Our findings are consistent with previous research on audiovisual simultaneity judgment tasks, where the authors showed that lower beta power over the scalp preceded visual-then-auditory sequences perceived as simultaneous trials (Yuan et al., 2016). Moreover, prestimulus lower beta band power has been associated with better sensory encoding (Griffiths et al., 2019), which might lead to illusory perception in case of incongruent AV stimulus input (for review, see Keil and Senkowski, 2018). Studies on rubber hand illusion have shown reduced central alpha and beta band power before illusory percept (Evans and Blanke, 2013; Rao and Kayser, 2017). Interestingly, however, our results deviate from multisensory illusion studies where they have reported a higher prestimulus beta power before the illusory percept (Keil et al., 2012; Keil and Senkowski, 2018; Kaiser et al., 2019). These studies have looked at beta power without removing the 1/*f* power (aperiodic) component, which might indicate that the aperiodic component of the power spectrum majorly drives the power in the prestimulus duration and separating the periodic power from the aperiodic component brings out the true nature of oscillatory activity (Monto et al., 2008; Becker et al., 2018; Donoghue et al., 2020b). Taking all these observations together, we propose that a lower periodic beta power in the frontal and occipital regions drives McGurk illusory percept instead of the higher beta power. Moreover, we also observed that lower peak beta center frequency (CF) in the occipital regions predicted illusory responses (Fig. 5*E*). These peak beta frequencies might be modulating audiovisual attention shift along with peak alpha center frequencies especially in the occipital region. However, looking at prestimulus cross-frequency coupling (CFC) is beyond the scope of this study.

Interestingly, our logistic mixed-effects model predicted significantly higher gamma bandwidth (BW) activity, with extreme evidence (BF > 100) in the frontal and temporal regions prior to illusory perception (Fig. 5*B*,*F,G*), while the role of bandwidth in multisensory perception remains rather underexplored. However, a review by Lowet et al. (2022) suggests that variability in gamma bandwidth reflects changes in neural synchronization driven by different cognitive demands, underscoring the adaptability of neural circuits to external stimuli. Based on this premise, we hypothesize that gamma band synchronization between frontal and temporal regions undergoes distinct changes before illusory perception compared with nonillusory conditions. However, examining the nature of this synchronization falls beyond the scope of the current study. Overall, the periodic activity pattern of high theta and low alpha power (in frontal, central, and occipital regions), alongside low beta (in frontal and occipital region) and high gamma power (over frontal and temporal regions), suggests a shift in attention modulation in the prestimulus duration. And, this shift appears to favor visual processing, thereby biasing perception toward the illusory interpretation of the upcoming McGurk stimulus.

### Decreased aperiodic offset and exponent parameters predicts response to the illusory speech sound perception

The aperiodic component of the power spectrum (offset and exponent) has been associated with modulations in cognitive states (He et al., 2010; Podvalny et al., 2015), aging (Voytek et al., 2015; Donoghue et al., 2020b; Thuwal et al., 2021), and the excitation/ inhibition (E/I) balance of local neuronal populations (Manning et al., 2009; Gao et al., 2017; Waschke et al., 2021; Chini et al., 2022). In our sensor-level analysis, we found a significant increase in offset values across the scalp (Fig. 4*E*) and flatter exponent over temporal and occipital sensors before the illusory percept (Fig. 4*F*). In contrast, our logistic mixed-effects models estimated that lower prestimulus offset and flatter exponent from parietal, central, and occipital electrodes predicted the illusory response to the upcoming McGurk stimulus (Fig. 5*C*,*D*,*E*). This apparent discrepancy reflects the distinct questions addressed by the two analyses. The sensor-level LMER captures average group-level differences, highlighting a general shift toward increased cortical excitability before illusion, while the logistic model leverages trial-by-trial variability to reveal that, within this elevated state, local reductions in aperiodic features are most predictive of illusory perception in specific regions. These findings suggest that both global and local aperiodic fluctuations shape multisensory outcomes, underscoring the complex, potentially nonlinear relationship between background neural activity and perceptual experience (He, 2014; Donoghue et al., 2020b).

Moreover, the offset parameter is often referred to as signal's baseline or “background noise,” which is positively correlated to spontaneous neural spiking (Manning et al., 2009; Miller et al., 2012). Therefore, our results on decreased offset value before illusory perception could reflect a decreased spontaneous neuronal population spiking activity over the central, parietal, and occipital regions, potentially indicating on a selective cortical state that leads to illusory percept. This could further be systematically tested by tracking population firing rate using computational models of neural masses with the excitation/inhibition (E/I) balance of local neuronal populations (Gao et al., 2017; Keil and Senkowski, 2017; Kumar et al., 2020) which is beyond the scope of the current study.

Furthermore, the exponent parameter refers to the steepness of exponential power decay with increasing frequencies, which is associated with underlying synaptic currents and reflects the underlying E/I balance of neural networks. A flatter exponent—as observed prior to McGurk illusory perception—suggests increased arrhythmic background neuronal firing which is thought to be driven by increased E/I ratio (Voytek et al., 2015; Gao et al., 2017; Lendner et al., 2020). However, the extant literature does not shed a clear mechanistic understanding of this phenomenon but rather provides a mixed understanding of this correlation in relation to higher-order cognitive processing. For instance, Wiltshire et al. (2017) have reported that spectral slope becomes more steep with increasing demands on externally oriented attention in a given task. Other studies have reported flatter slopes in association with higher state of consciousness (Miskovic et al., 2019; Lendner et al., 2020), in rest to task state transitions (Podvalny et al., 2015), and in neuromodulatory processing of uncertainty (Kosciessa et al., 2021). Considering all these inferences, we propose that in case of multisensory McGurk perceptual task, a flatter exponent before illusory percept over central, parietal, and occipital sensors reflects a shift in cortical dynamics toward a more excitable state. Such a shift might arise under conditions with high uncertainty like the prestimulus state, where cortical networks transition from a rhythmically dominated regime to a predominantly aperiodic (or arrhythmic) state. This high excitable and arrhythmic state might be better suited for integrating incongruent McGurk stimulus leading to illusory perception. However, these interpretations remain under active investigation, and therefore our findings should be viewed as consistent with, but not definitive of, these proposed mechanisms.

### Interactions between mean periodic power and aperiodic exponent predict multisensory speech sound illusion

Significant interactions between mean periodic power (aperiodic adjusted) and aperiodic exponent during prestimulus intervals also predicted subsequent responses to the McGurk illusion on a trial-by-trial basis. Specifically, we observed that decreased occipital theta power and increased occipital and parietal gamma power were associated with a higher likelihood of illusory perception when the aperiodic exponent was steeper (Fig. 7*A*,*D*). Similarly, decreased global beta power predicted illusory responses in the context of a steeper exponent (Fig. 7*C*), while decreased alpha power and a steeper exponent individually predicted illusory perception, though their interaction was not significant (Fig. 7*B*). These results align closely with studies linking the aperiodic exponent to the underlying excitation–inhibition (E/I) balance in cortical circuits (Voytek et al., 2015; Gao et al., 2017). A steeper exponent is indicative of a more inhibition-dominated (lower E/I) state, which has been associated with reduced population spiking (Miller et al., 2009; Voytek and Knight, 2015; Zhou and Yu, 2018; Medel et al., 2023; Diachenko et al., 2024). Moreover, in our study, the decreased low-frequency (theta, beta) power in conjunction with a steeper exponent predicts illusory percept is consistent with previous reports that low-frequency power is correlated with population spiking (Miller et al., 2007, 2009; Ball et al., 2008; Voytek and Knight, 2015), suggesting that reduced synchronous low-frequency activity, in our case, occipital theta and global beta, associated with steeper exponent (less population spiking) may facilitate prestimulus oscillatory dynamics that shapes McGurk illusory perception.

Moreover, we also observed an increase in posterior (occipital and parietal) gamma power predicted the illusory perception when the exponent was steeper. These observations support the notion that gamma activity, often linked to local cortical excitation and perceptual binding, may be more functionally relevant in a subcritical (or inhibition-dominated) state (Cohen and Maunsell, 2011; Harris and Thiele, 2011; Waschke et al., 2021). This interplay between aperiodic and mean power highlights the importance of considering both oscillatory and aperiodic neural dynamics in understanding perceptual outcomes. Our interaction model results suggest that a transient shift toward a more inhibited, and more ordered neural state, indexed by a steeper aperiodic exponent and selective modulation of mean power (lower occipital theta, lower global beta, and higher posterior gamma power), may facilitate the perceptual binding processes underlying the McGurk illusion. Overall, we speculate that while different oscillatory features govern distinct cognitive functions (Haegens et al., 2014; Mierau et al., 2017; Scally et al., 2018), they also interact significantly to modulate individual participants’ predisposition of prestimulus brain state over individual trials, which leads to experiencing illusory or nonillusory perception in case of McGurk stimulus.

Taken together, in this study we have attempted to understand the relationship between prestimulus oscillatory activity and aperiodic activity in subsequently perceiving the McGurk illusion on a trial-by-trial basis. We have demonstrated that aperiodic activity (especially steeper aperiodic exponent) greatly influences spectral power at both unisensory (temporal and occipital) and higher cognitive areas (like parietal and frontal) in shaping perceptual response. We believe that this interaction accounts for intertrial variability which is quintessentially observed in McGurk paradigms. Therefore, this study opens a new avenue for addressing the role of prestimulus aperiodic activity in influencing low- and high-frequency band oscillations across relevant brain areas. Moreover, by parameterizing the power spectrum one can study the modulation effect of one true oscillatory activity from another in the context of multisensory speech perception.

## Synthesis

Reviewing Editor: Anne Keitel, University of Dundee Decisions are customarily a result of the Reviewing Editor and the peer reviewers coming together and discussing their recommendations until a consensus is reached. When revisions are invited, a fact-based synthesis statement explaining their decision and outlining what is needed to prepare a revision will be listed below. The following reviewer(s) agreed to reveal their identity: NONE.

The revised manuscript has been re-assessed by the two original reviewers. While some aspects of the manuscript have improved, both reviewers felt that the revisions were overall not sufficient to alleviate all their concerns. The main points were:

- Some statistical decisions unclear or problematic

- Interpretation potentially not in line with results

- Inaccuracies with references

Therefore, the decision of a major revision ("Revise and Re-review") has been reached. The unabridged reviewer comments, including more points than mentioned here, are below. I strongly recommend to carefully address each comment in a point-by-point response and add the requested analyses. Please let me know if you need more time for a thorough revision, an extension is no problem.

-----

Reviewer #1

Advances the Field (Required)

Integrating oscillatory and aperiodic components of neural activity in the analysis of multisensory integration has not been done before.

Statistics

Pooling trials across participants obscures interindividual differences. The multiple data analyses are not described in sufficient detail, and the reason for the analyses is missing.

Extended Data

The extended data comprises additional tables and figures to describe the findings. The results are rather confusing and the extended data des not alleviate this.

Comments to the Authors (Required)

In the current manuscript, the authors aim to disentangle the influence of aperiodic and periodic (i.e., oscillatory) neural activity on multisensory perception. The latter is operationalized in the form of the McGurk illusion. The current manuscript is the first revision of a previously submitted manuscript. In the review process, the authors took great care to address the issues raised by the reviewers. Unfortunately, not all concerns could be resolved. In general, the manuscript is still rather unstructured, and it is not clear what the single analyses are contributing to answering the research question. As a result, I don't think the current results warrant a discussion yet. Therefore, I don't comment on this section.

Overall, I suggest paying more attention to clear descriptions. Often, shorter sentences without intermixing different findings can be helpful. Similarly, I suggest a careful review of grammar, the use of singular and plurals, and punctuation.

Please find a more detailed discussion of major and minor issues below.

Major:

Introduction: As mentioned with respect to the previous version of the manuscript, I urge the authors to be more careful and precise with the use of references. Just one example: The paper by Buzsáki et al., 2012 (like the paper by Ray and Maunsell, 2011) neither refers to the relationship between aperiodic offset and neuronal spiking patterns, nor to the relationship between aperiodic exponent and E/I-balance. Similarly, the paper by Engel et al., 2001 does not mention aperiodic components. Because of this, I can't trust the authors' assessment of the papers mentioned in this newly added paragraph to the introduction, and I will ignore the information provided here, and suggest a careful revision of the entire introduction to only deliver relevant and correct information.

Statistical analysis: As mentioned with respect to the previous version of the manuscript, I suggest thinking about the reason for each analysis and what it contributes to answering the research question of the current manuscript. As mentioned before, I have strong concerns about the multiple parallel analyses, and the answer in the rebuttal has not alleviated these concerns. In this analysis, pooling trials from different participants is highly problematic. I can follow the argument that including the subject ID as random effect in the linear model analysis described below takes care of the between-subject variability. However, in the unpaired test between illusory and non-illusory trials, this individual variability is not considered. I would like to advise the authors to carefully think about what this analysis contributes to the overall analysis.

Correlation analysis: Thank you for including this information in the methods section. However, I don't think the information provided here is sufficient. Please state how the mean oscillatory power was obtained. I count 18 data points for illusion and non-illusion trials in figure 6, so power and aperiodic activity must be computed within each participant. However, there are some participants who have no illusion or non-illusion trials, as can be seen in figure 2.

Interactions between aperiodic parameters and oscillatory activity predicts perception to McGurk stimulus: In the theta interaction model, you mention a three-way interaction between power, exponent and region. However, the effect of the region is not further described, but only the two-way interaction. Where is this effect coming from? The same is true for the gamma interaction model.

Minor:

Abstract: The description "frontal-occipital" does not make much sense. I suggest using "frontal and occipital".

Abstract: I suggest splitting the description of the power effects from the associated statistical evidence. The sentence stub "with extreme evidence in the frontal and temporal regions" does not make much sense without a qualifier for what this is extreme. Moreover, please be more precise in the description of the effects: "oscillations" are characterized by power, phase, and frequency. I therefore suggest using "power" here.

Predicting response to the McGurk trials from prestimulus parameterized power: Thank you for the explanation of the 2639 data points. Unfortunately, this explanation is missing from the paper, so the uninformed reader can't follow this. Moreover, because you performed this analysis separately for the 6 sensor regions, I don't think the information provided in brackets is clear. I suggest including the explanation provided in the rebuttal regarding the trial number instead of this confusing statement.

Variability in illusory percept observed within and between participants: What does the sentence "the paired sample t test revealed no significance" refer to? I assume you compared the response probability between conditions. If so, I would explicitly add this to the results of the t-test (e.g.: "the paired sample t test revealed no significant difference in the number of illusion and non-illusion trials").

Periodic and aperiodic oscillations of the whole brain differ between illusory and non illusory McGurk trials: First, please note that the aperiodic component of neural activity is not an oscillation. I therefore suggest changing the section title. Second, can you please add the degrees of freedom to the report of t-values. This would allow appreciating the number of observations included here. Please also keep the number of decimals and the reporting of effect sizes consistent. Similar, why did you perform non-parametric tests for the aperiodic parameters? This is not described in the methods section. Not finding a significant difference in offset and exponent also does not allow a statement such as "We found lower...". This also does not allow a statement such as "These results imply that there are differences in prestimulus aperiodic activity...". In general, I still don't think it is a valid approach to pool trials across participants here and in the examination of spatial topographies.

Prestimulus periodic and aperiodic parameters across different sensor regions can predict the response to the upcoming McGurk stimulus: A statement such as "Aperiodic offset and exponent parameters from central, parietal, and occipital regions were found to be better predictors to predict response to upcoming McGurk stimulus" would require stating a comparison (i.e., "better" than what) and a statistical test to qualify such a comparison.

Signed: Julian Keil

-----

Reviewer #2

Advances the Field (Required)

The authors include two interesting features to their analysis of interindividual variability in multisensory illusory perception: use of statistical methods that allow to evaluate variability across subjects and trials and, on the other, they incorporate aperiodic activity as a feature of interest.

Statistics

It was not clear for me how the multiple comparison correction was applied.

Extended Data

They have provided some images and tables that are relevant for the study

Comments to the Authors (Required)

I have read the revised version of the manuscript "Prestimulus Periodic and Aperiodic Neural Activity Shapes McGurk Perception". The authors addressed several of the points raised in the previous review but, in my opinion, the manuscript is not ready for publication yet. I summarize my concerns below.

-Interpretation

This point is crucial. According to my understanding, the discussion and results are inconsistent. In the previous review I asked the authors to check for a bias towards the visual modality, as they claimed, or to remove this claim from the discussion. The authors chose to reformulate the statement in the discussion, and I wonder why they did not perform a simple t-test to check whether subjects displayed a preference towards visual stimulus in the incongruent non-illusory trials. More importantly, this statement does not fit with what is reported in the paper I will copy below the paragraphs that make consider there is an inconsistency in the results. In the Methods section, the authors write:

"Each participant viewed 600 trials of videos of a male annotator (matched by the geographic region and demographic background of the participants) articulating the syllables /pa/, /ta/, and /ka/. Participants were instructed to report their subjective perceptions of the audio-visual (AV) stimuli. The experiment included four types of AV stimuli: three congruent (audio syllables matching video articulation) syllables /pa/, /ta/, and /ka/, and one McGurk (audio-visual mismatch) syllable (auditory /pa/ with visual /ka/), which created the illusion of syllable /ta/."

So, according to this paragraph, /pa/ corresponds to the auditory part of the McGurk stimulus. Then, behavioural results are reported (Results section):

"For incongruent McGurk condition, illusion was perceived in 58.38% (SD = 32.5) of trials, whereas, unisensory /pa/ was perceived in 37.39% (SD = 31.94) of trials (Figure 2B (ii))."

For clarity, the modality of /pa/ should be stated. According to the methods, /pa/ is auditory. Therefore, the results describe that in the incongruent trials 58.38% are illusory, 37.39% are perceived as the auditory modality (/pa/), which would leave 4.23% trials perceived as the visual modality.

Finally, in the discussion, the authors talk about a possible bias towards the visual sensory input:

"While we did not directly measure behavioral indices of visual bias in our participants, these studies, however, suggests that the lower prestimulus alpha observed in our study (Figure 3A) specifically in the occipital sensors (Figure 4) prior to the McGurk illusion might reflect a perceptual bias towards visual sensory input."

Given the numbers reported in the behavioral section, if there is a bias, wouldn't the authors interpret, according to the data they report, that the bias would be towards the auditory modality? This needs to be clarified. As it is clear from the methods that the authors have the data required to test for a potential bias (preferred modality in the non-illusory incongruent trials), they should do the test or remove the paragraph from the discussion.

-Background

The authors have now improved the introduction, adding information about aperiodic activity and the clarifying the relevance of their approach for the study of McGurk illusion. However, there are still some gaps that need to be filled.

The authors seem to assume that brain oscillations and aperiodic activity result from different neural processes. This is a valid hypothesis, but still they should acknowledge that there is an alternative work hypothesis: that aperiodic activity and brain oscillations coexist and can result from same neural populations. See, for instance: Gireesh, E. D. &Plenz, D. Neuronal avalanches organize as nested theta- and beta/gamma-oscillations during development of cortical layer 2/3. Proceedings of the National Academy of Sciences 105, 7576-7581 (2008).

The justification for including bandwidth provided in the introduction is feeble, just because a parameter has not been explored, this does not mean that is relevant. However, in the discussion the authors do provide an interpretation that justifies the interest of evaluating bandwidth. I suggest that the authors mention the review of Lowett et al (2022) already in the introduction to strengthen the motivation of including bandwidth in the analyses.

-Multiple comparison correction,

I am surprised that the p-values after multiple comparison correction remain completely unchanged. Could the authors explain how this was performed? I have the impression that they applied the correction separately for each model, am I right? I believe I was not clear in my previous review, but I would be more in the line of correcting for the different tests performed (for instance, different frequency bands evaluated).

-Parametrized power

The authors claim that they separate aperiodic power from oscillatory activity, but this is not completely true. The authors do not check, according to the report, whether the activity that remains in a given frequency band corresponds to an oscillation or not (See, for instance: https://onlinelibrary.wiley.com/doi/10.1111/ejn.15829). Even if aperiodic contribution is removed from the data, this does not imply that the remaining activity is periodic. It would be important that the authors acknowledged this limitation in their study and the implications this could have for the interpretation of the results. For instance, according to Figure 3 and Extended data figure 3.1 there is no peak in theta activity (either in parametrized and non parametrized power), and therefore the differences observed in theta could be due to shifts in alpha peak, or changes in the bandwidth of alpha, as one could interpret from the graph.

Following citation:

Fernandez M, L., Torralba, M., &Soto‐Faraco, S. (2018). Theta oscillations reflect conflict processing in the perception of the McGurk illusion. European Journal of Neuroscience, 48(7), 2630-2641. https://doi.org/10.1111/ejn.13804

Should be

Morís-Fernández L., Torralba, M., &Soto‐Faraco, S. (2018). Theta oscillations reflect conflict processing in the perception of the McGurk illusion. European Journal of Neuroscience, 48(7), 2630-2641. https://doi.org/10.1111/ejn.13804

-Data availability statement

The authors need to provide a valid link to the data and code for the review process:

"The raw EEG data used in this study are available from the corresponding authors upon reasonable request. However, the processed EEG data and all the relevant codes used for subsequent analysis are available in the provided link (URL is redacted)."

## Author Response

Response to the Reviews eN-NWR-0431-24R1 We sincerely thank the editor and reviewers for their feedback and important suggestions to further improve our manuscript. We are thankful to the editor and both reviewers, as their comments have significantly improved the original submission. In the second revision, we have addressed all their comments in a point-by-point fashion. In the current manuscript, all the revised text are highlighted in yellow. The excerpts from the main manuscript are in italics with line and page numbers mentioned wherever applicable. Please see below the detailed outline of our point- by-point responses in the same order the comments appeared.

Reviewer 1 (Major) 1. "Introduction: As mentioned with respect to the previous version of the manuscript, I urge the authors to be more careful and precise with the use of references. Just one example: The paper by Buzsáki et al., 2012 (like the paper by Ray and Maunsell, 2011) neither refers to the relationship between aperiodic offset and neuronal spiking patterns, nor to the relationship between aperiodic exponent and E/I-balance. Similarly, the paper by Engel et al., 2001 does not mention aperiodic components. Because of this, I can't trust the authors' assessment of the papers mentioned in this newly added paragraph to the introduction, and I will ignore the information provided here, and suggest a careful revision of the entire introduction to only deliver relevant and correct information." Response: We thank the reviewer for highlighting the importance of appropriate referencing and for pointing out specific instances where some citations in our Introduction did not directly support the claims made. In response, we have carefully reviewed the entire Introduction and revised the citations to ensure that each reference accurately corresponds to the claim it supports. Specifically, we have:

A. Removed references (like Buzsáki et al., 2012; Ray &Maunsell, 2011; and, Engel et al., 2001) which do not directly address the relationship between aperiodic offset/exponent and neuronal spiking or excitation-inhibition (E/I) balance, and do not discuss aperiodic components.

B. Added appropriate references that directly support the relevant claims. For example, we now cite Manning et al., (2009) and He B., (2014) for the association between aperiodic offset and neuronal spiking, and Gao et al., (2017) and Donoghue et al., (2020b) for the relationship between aperiodic exponent and E/I balance. We also added He B., (2014) and Donoghue et al., (2020b) for general background on aperiodic (1/f) activity.

All the changes in the citations are highlighted in the manuscript. Refer to page 3 of the manuscript.

2. "Statistical analysis: As mentioned with respect to the previous version of the manuscript, I suggest thinking about the reason for each analysis and what it contributes to answering the research question of the current manuscript. As mentioned before, I have strong concerns about the multiple parallel analyses, and the answer in the rebuttal has not alleviated these concerns. In this analysis, pooling trials from different participants is highly problematic. I can follow the argument that including the subject ID as random effect in the linear model analysis described below takes care of the between-subject variability. However, in the unpaired test between illusory and non- illusory trials, this individual variability is not considered. I would like to advise the authors to carefully think about what this analysis contributes to the overall analysis." Response: We sincerely thank the reviewer for raising a valuable point regarding the statistical analysis applied in our study, particularly about pooling trials across participants and the concern regarding handling of individual variability in the unpaired test applied between illusory and non-illusory trial conditions. With regards to the unpaired test performed in the previous version of our study, our goal was to address the core research question of whether there is a significant difference in power between the two trial conditions before McGurk perception. However, we acknowledge the issue raised regarding pooling trials from different participants before performing the test. We understand that while pooling increases statistical power, it can obscure individual differences if not properly controlled, which in the previous version of the manuscript was not accounted for, which might have inflated Type I error rates or obscure true effects due to inter-individual differences. In the current version of the manuscript, we have accounted for inter-subject variability while comparing group-level differences. Below are the excerpts from the current manuscript accounting for and reporting the new and improved group-level differences: (Line 208 - 247, Page 10-12; Materials and Method section) "Statistical analysis At the group-level To examine group-level differences in spectral power (both prestimulus and poststimulus) between illusory (/ta/) and non-illusory (/pa/) trials across frequency bands, a repeated- measures analysis of variance (ANOVA) was performed using MATLAB's ranova function. The analysis employed a 2 (Condition: /ta/, /pa/) x 4 (Frequency: Theta, Alpha, Beta, Gamma) within-subject design. For each participant, mean power values were computed for each frequency bands and condition, and these were fit into the repeated measures ANOVA model. One participant with 100% illusory /ta/ McGurk susceptibility was excluded from the group-level pairwise analysis. Sphericity was evaluated using Mauchly's test, and Greenhouse-Geisser corrections for p-values (pGG) were applied when the assumption of sphericity was violated. Partial eta-squared (η2) was calculated to estimate effect sizes for main and interaction effects. Post-hoc pairwise comparisons between condition within each frequency band were performed using Tukey HSD test for multiple comparison corrections. To further dissociate condition effects on periodic power and aperiodic components, a similar repeated-measures ANOVA was conducted on parameterized spectral features. This analysis used a 2 (Condition: /ta/, /pa/) × 6 (Component: mean theta, alpha, beta, gamma power; aperiodic offset, aperiodic exponent) within-subjects design. The same procedures for sphericity assessment, correction, effect size estimation, and post-hoc testing were applied as in the power spectral analysis.

At the sensor-level To assess differences between illusory /ta/ and non-illusory /pa/ conditions at the sensor level, we used a mass univariate linear mixed-effects regression (LMER) approach implemented in the lme4 package (v1.1.32; Bates et al., 2015) in R (R Development Core Team, 2022). A separate model was fit at each sensor, with the spectral feature of interest- mean periodic power across frequency bands, aperiodic offset, or aperiodic exponent- modeled as a function of Condition (fixed effect), while including Subject ID as a random intercept to account for repeated measures and inter-individual variability. The model formula was:

Values ~ Condition+(1|Subject ID) (1) where, Values denoted the spectral feature of interest at each sensor and trial. Condition reflected the two response conditions- illusory /ta/ and non-illusory /pa/. To control for multiple comparisons, we implemented permutation testing at the sensor-level. For each sensor, the condition labels were randomly permuted within each subject (preserving the within-subject structure) to generate a null distribution of the t-value estimated for the condition effect (Maris and Oostenveld, 2007; Visalli et al., 2024). This process was randomized for 1000 iterations per sensor. The observed t-value was compared to the null distribution to compute a two-tailed permutation p-value for each sensor. To further control the false discovery rate arising from multiple statistical tests across all sensors and spectral features, we applied the Benjamini-Hochberg procedure to jointly adjust the permutation p-values (Benjamini &Hochberg, 1995). Sensors and features with FDR-corrected p- values below the significance threshold (alpha = 0.05) were considered as significant, ensuring robust inference across the entire set of comparisons." (Line 377-410, Page 18-19; Results section) "No group-level differences observed in periodic power and aperiodic activity between illusory and non-illusory McGurk trials A repeated measures ANOVA with a 2 (Condition: /ta/ and /pa/) x 4 (Frequency: Theta, Alpha, Beta, Gamma) within-subjects design was computed to analyse power spectrum differences (PSDs) between illusory and non-illusory response conditions. During prestimulus duration, the analysis revealed no significant interaction between Condition and Frequency (F(3,48) = 0.239, pGG = 0.868, partial η2 = 0.015). However, post-hoc pairwise comparison with Tukey-HSD multiple correction revealed significantly higher beta power (mean difference = 0.339, 95% CI = [0.015, 0.662], p = 0.041, Cohen's D = 0.539) and higher gamma power (mean difference = 0.575, 95% CI = [0.229, 0.921], p = 0.003, Cohen's D = 0.854) before illusory perception (Figure 3A (a)). For poststimulus duration, the repeated measures ANOVA revealed no significant interaction between Condition and Frequency (F(3,48) = 0.529, pGG = 0.571, partial η2 = 0.032). Post-hoc tests revealed no significant condition differences across frequency bands (Figure 3A (b)). Consistent with previous findings by Keil et al. (2012), we observed higher prestimulus beta-band power associated with illusory perception; however, we did not observe a reduction in theta power following illusory perception.

We further examined the group-level differences in periodic power and aperiodic component estimated by parameterizing the power spectrum using SpecParam (FOOOF) model (Donoghue et al., 2020b). The periodic oscillations were computed after removing the aperiodic component in linear space from untransformed power spectrum from trials where peaks were detected after fitting the FOOOF model. A repeated measures ANOVA with a 2 (Condition: /ta/ and /pa/) x 6 (Component: mean theta, alpha, beta, gamma power; aperiodic offset, aperiodic exponent) within-subjects design was computed to analyse differences in periodic power and aperiodic component between illusory and non-illusory response conditions. We observed no significant interaction between Condition and Component for either the prestimulus (F(5,80) = 0.343, pGG = 0.632, partial η2 = 0.021; Figure 3B) or poststimulus duration (F(5,80) = 1.548, pGG = 0.227, partial η2 = 0.090; Extended Data Figure 3-1). Moreover, post-hoc tests also revealed no significant pairwise condition difference for aperiodic components (offset and exponent) and periodic power across frequency bands. These findings emphasize the importance of separating aperiodic activity from periodic oscillations to accurately characterize the spectral changes associated with the McGurk illusory percept. Importantly, the absence of group-level effects prior to McGurk perception suggests that individual differences in susceptibility may obscure perception-related neural dynamics, making inter-trial analyses a more sensitive approach to uncover the underlying mechanisms before McGurk perception. We then proceeded to capture the trial-wise variability at the sensor space. (Figure 3) (Figure Legend: Line 1121-1130, Page 49) Figure 3: Grand-averaged power spectral densities (PSDs) and parameterized power distributions. (A) Group-averaged PSDs computed from subject-specific spectra, averaged over all sensors and trials within each participant. Mean power spectra are shown for illusory (red) and non-illusory (blue) McGurk trials, with shaded areas representing the standard error of the mean (SEM), separately for the (a) prestimulus and (b) poststimulus periods. (B) Prestimulus power distributions derived from parameterized spectra. Panel (a) shows grand-averaged periodic (oscillatory) power, averaged over all sensors and trials within each participant for illusory (red) and non-illusory (blue) conditions, with SEM shading. Panel (b) displays box-and-whisker plots overlaid with density distributions for the aperiodic parameters-offset (top) and exponent (bottom)-averaged over sensors and trials within each participant, grouped by perceptual condition (red = illusory; blue = non-illusory). (Extended Data Figure 3-1) (Figure Legend: Line 1179-1183, Page 51-52) "Extended Data Figure 3-1: Parametrized power distributions after perception of McGurk trials indicating inter-individual (or group-level) variability. (A) Poststimulus periodic power distributions for the illusory (red) versus non-illusory (blue) McGurk trials with SEM as shaded region. (B) Box and whisker plots with density distributions for comparisons between poststimulus aperiodic offset (above) and exponent (below), by response conditions (red - illusory, and blue - non-illusory)." (Line 412-437, Page 19-20; Results section) Spatial topographies of prestimulus periodic power and aperiodic component show distinct brain regions involved in illusory and non-illusory perception To investigate differences in prestimulus spectral activity preceding illusory /ta/ and non- illusory /pa/ perceptions, we compared trial-wise spatial topographies of periodic power (theta, alpha, beta, gamma) and aperiodic components (offset, exponent) using mass univariate linear mixed-effects models with permutation-based correction (see Materials and Methods section for details). Regression models revealed significant differences across a distributed set of scalp sensors after FDR correction (p < 0.05). Band-specific patterns revealed distinct topographical profiles. A higher mean theta power was observed over frontal F4 and F6 sensors before illusory perception (Figure 4A). A lower mean alpha power was observed over frontal, frontal-central, and frontal-temporal sensors; whereas a higher alpha power over central sensors (C5, C1, C6) were observed before illusion (Figure 4B). A lower beta power was observed across scalp before McGurk illusory perception (Figure 4C). A higher gamma power over frontal, central, parietal, occipital, and right temporal sensors were observed; and a lower gamma power was observed over left temporal sensors before illusory perception (Figure 4D). For the aperiodic offset, a higher t-value was observed across scalp (Figure 4E). And, finally lower aperiodic exponent was observed across temporal and occipital sensors and a higher exponent over FC3 and FC5 sensors was observed before illusory perception (Figure 4F). Taken together, these spatial topographical distributions of mean periodic power and aperiodic component, indicate distinct prestimulus brain states preceding the perception of an impending McGurk stimulus. These differences are evident not only in the unisensory perceptual regions like the occipital and temporal sensor regions but also in higher cognitive processing regions, such as the frontal, central, and parietal areas. So, to further understand how changes in these prestimulus spectral features relate to varying perception to McGurk stimulus, we further divided the whole scalp into six distinct sensor clusters covering bilateral frontal, central, parietal, occipital, and unilateral left temporal and right temporal sensors (see Table 1 for details). And then, fitted logistic mixed-effect models across the entire brain and different sensor regions." (Figure 4) (Figure Legend: Line 1132-1138, Page 49-50) Figure 4: Topographic distribution of t-values from linear mixed-effects models for periodic and aperiodic spectral components. Topoplots depicting t-values from linear mixed-effects models comparing illusory (/ta/) and non-illusory (/pa/) McGurk trials for mean periodic power across (A) theta, (B) alpha, (C) beta, and (D) gamma frequency bands, as well as aperiodic components: (E) offset and (F) exponent. The color bar indicates the corresponding t-values. White-marked sensors denote statistically significant differences between conditions. Periodic power was computed by subtracting the aperiodic fit from the original (untransformed) power spectral densities in linear space. (Line 547-549, Page 25; Discussion section) "Four key findings emerged from this study: (1) No significant differences in power across frequency bands were observed across participants after adjusting for the aperiodic component prior to McGurk trials;" (Line 565-569, Page 26; Discussion section) This suggests that the previously reported beta effects may have been influenced by underlying aperiodic activity, highlighting the importance of disentangling aperiodic and periodic components when interpreting power spectral differences related to McGurk perception. In contrast, our trial-wise sensor-level analysis revealed significantly higher prestimulus theta power over frontal sensors prior to illusion perception (Figure 4A)." "Moreover, lower prestimulus alpha band power has been associated with higher cortical excitability (Mathewson et al., 2009, 2011; Romei et al., 2008, 2010; Samaha et al., 2017). This means that at regions with high excitability (lower alpha power), the threshold of activation of the underlying neural population is lowered. Thus, improving the neural processing of sensory input leading to better perception, which in case of incongruent McGurk stimulus sometimes may lead to illusory percept (Van Dijk et al., 2008; Jensen et al., 2012; Lange et al., 2014). In our study, we observed lower prestimulus alpha periodic power in the frontal and central sensor regions prior to illusory perception (Figure 4B). This might reflect increased excitability or attentional engagement of higher-order networks that might be critical for multisensory integration in McGurk illusion (Foxe &Snyder, 2011; Haegens et al., 2012; Sadaghiani &Kleinschmidt, 2016). Fluctuations in frontal and central alpha power have also been linked to top-down modulation of attention and preparatory states that influence perceptual outcomes (Jones et al., 2010; Klimesch, 2012; Samaha et al., 2015a). Notably, while we did not find significant occipital alpha power differences at the sensor level, our analysis of peak alpha frequency (PAF) in the occipital region (Figure 5E) revealed that lower PAF predicted illusory percepts that is consistent with previous findings that occipital PAF modulates audiovisual integration and susceptibility to multisensory illusions (Cecere et al., 2015; Keil &Senkowski, 2017; Samaha &Postle, 2015b; Venskus &Hughes, 2021). Taken together, these results suggest that lower prestimulus alpha power in frontal and central regions, along with lower occipital PAF, may jointly facilitate the attentional and integrative processes that bias perception toward the McGurk illusion. This aligns with the view that alpha oscillations reflect both local excitability and large-scale network dynamics that shape multisensory perceptual experiences (Foxe &Snyder, 2011; Sadaghiani &Kleinschmidt, 2016)." (Line 644-648, Page 29; Discussion section) "Overall, the pattern of high theta and low alpha power (in frontal, central, and occipital regions), alongside low beta (in frontal and occipital region) and high gamma power (over frontal and temporal regions) suggests a shift in attention modulation in the prestimulus duration. And, this shift appears to favor visual processing, thereby biasing perception toward the illusory interpretation of the upcoming McGurk stimulus." (Line 656-667, Page 30; Discussion section) "We found a significant increase in offset values across the scalp (Figure 4E) and flatter slopes of 1/f background activity (exponent) over temporal and occipital sensors before the illusory percept (Figure 4F). In contrast, our logistic mixed-effect models estimated that lower prestimulus offset and flatter exponent from parietal, central, and occipital electrodes predicted the illusory response to the upcoming McGurk stimulus (see Figure 5C, 5D, and 5E). This apparent discrepancy reflects the distinct questions addressed by the two analyses. The sensor-level LMER captures average group-level differences, highlighting a general shift toward increased cortical excitability before illusion, while the logistic model leverages trial-by-trial variability to reveal that, within this elevated state, local reductions in aperiodic features are most predictive of illusory perception in specific regions. These findings suggest that both global and local aperiodic fluctuations shape multisensory outcomes, underscoring the complex, potentially non-linear relationship between background neural activity and perceptual experience (Donoghue et al., 2020b; He, 2014)." 3. "Correlation analysis: Thank you for including this information in the methods section.

However, I don't think the information provided here is sufficient. Please state how the mean oscillatory power was obtained. I count 18 data points for illusion and non-illusion trials in figure 6, so power and aperiodic activity must be computed within each participant. However, there are some participants who have no illusion or non-illusion trials, as can be seen in figure 2." Response: We have now updated the Methods section and Figure 6 legend to include more details about the correlation analysis performed: section) "Correlation analysis The association between prestimulus mean aperiodic adjusted power (across frequency bands) and aperiodic parameters (offset and exponent) was examined using Spearman rank correlation. The mean aperiodic adjusted power was obtained by subtracting the aperiodic fit in linear space from the original untransformed spectrum (see Gyurkovics et al., 2021). Correlations were computed for illusory /ta/ and non-illusory /pa/ trial conditions separately. To compute these associations, channel and trial-averaged aperiodic adjusted power and aperiodic parameters was computed for each participant. Participants with zero trials in a given condition were excluded from that condition's correlation analysis." (Figure 6 Legend; Line 1155-1161, Page 51-52) "Figure 6: Association between aperiodic exponent and mean aperiodic adjusted power in the (A) Theta, (B) Alpha, (C) Beta, and (D) Gamma frequency bands before perception of illusory (red) and non-illusory (blue) McGurk trials averaged across all channels and trials for each participant. The aperiodic slope is represented on the x-axis and aperiodic adjusted power on the y-axis. The density of observations for each association plot is indicated on the margins for illusory (red) and non-illusory (blue) trial conditions. One participant contributed zero non-illusory trials, so the non-illusory condition includes N = 17." 4. "Interactions between aperiodic parameters and oscillatory activity predicts perception to McGurk stimulus: In the theta interaction model, you mention a three-way interaction between power, exponent and region. However, the effect of the region is not further described, but only the two-way interaction. Where is this effect coming from? The same is true for the gamma interaction model." Response: Thank you for your raising this important point regarding the three-way interaction between power, exponent, and sensor region in the theta and gamma interaction models. We agree that further elaboration would help clarify the nature of the regional effects. Below we have updated the Abstract, Results and Discussion section, and updated the Figure 7 of the manuscript to incorporate the new results: (Line 14-17, Page 1; Abstract section) "Our logistic mixed interaction models revealed that the aperiodic exponent and periodic power jointly influence the perception of upcoming McGurk stimuli. Specifically, a decrease in occipital theta and global beta power and an increase in occipital and parietal gamma power were associated with a steeper slope." (Line 507-530, Page 23-24; Results section) "In case of the theta interaction model, we found a significant three-way interaction between theta power X exponent X sensor regions (χ2 (5) = 18.822, p(adjusted) = 0.0066). This three-way interaction was most pronounced in the occipital sensor region (Log Odds= -0.082, 95% CI = -0.16 - -0.001, p = 0.038). The model revealed that when the exponent was steeper and as theta power lowered, the probability of illusory percept was higher (Figure 7A). For the alpha interaction model, no significant two-way interaction (mean alpha power X exponent, χ2 (1) = 0.3849, p(adjusted) = 0.6795) or three-way interaction (mean alpha power X exponent X sensor regions, χ2 (5) = 9.951, p(adjusted) = 0.1752) was observed. However, both mean alpha power and the exponent were individually significant predictors (mean alpha power: χ2 (1) = 25.9706, p(adjusted) = <0.0001; exponent: χ2 (1) = 15.3560, p(adjusted) = 0.0005). Figure 7B (left) illustrates that the probability of an illusory percept increases as alpha power decreases, while Figure 7B (right) shows that a steeper exponent is associated with a higher probability of an illusory percept. While the interaction was not statistically significant, this trend suggests a potential relationship between the predictors. For beta interaction model, there was a significant two-way interaction between beta power X exponent (χ2 (1) = 9.205, p(adjusted) = 0.0070). As depicted in Figure 7C, when the exponent was the steepest and as beta power lowered, the likelihood of perceiving the illusion increased. Finally, for gamma interaction model, a significant three-way interaction between gamma power X exponent X sensor regions (χ2 (5) = 21.586, p(adjusted) = 0.0025) was observed. The effect was most pronounced in the occipital (Log Odds = 0.19, 95% CI = 0.06 - 0.32, p = 0.0036) and parietal (Log Odds = 0.17, 95% CI = 0.05 - 0.29, p = 0.0041) sensor regions, where when the exponent was steeper and as the gamma power increased, the probability of illusory percept was higher (Figure 7D). Overall, these results suggest that when there is a steeper exponent, decreased theta and beta power were associated with better predicting the illusory response. Contrastingly, a higher gamma power associated with steeper exponent had a higher probability of perceiving the McGurk illusion." (Line 552-554, Page 25; Discussion section) "(4) The steeper aperiodic exponent associated with lower occipital theta, global beta, and higher posterior gamma power shape the perception of the upcoming McGurk stimulus." (Line 693-727, Page 31-33; Discussion section) "Interactions between mean periodic power and aperiodic activity predict multisensory speech sound illusion.

Significant interactions between mean periodic power (aperiodic adjusted) and aperiodic slope (or exponent) during prestimulus intervals, also predicted subsequent responses to the McGurk illusion on a trial-by-trial basis. Specifically, we observed that decreased occipital theta power and increased occipital and parietal gamma power were associated with a higher likelihood of illusory perception when the aperiodic exponent was steeper (Figures 7A and 7D). Similarly, decreased global beta power predicted illusory responses in the context of a steeper exponent (Figure 7C), while decreased alpha power and a steeper exponent individually predicted illusory perception, though their interaction was not significant (Figure 7B). These results align closely with studies linking the aperiodic exponent to the underlying excitation-inhibition (E/I) balance in cortical circuits (Voytek et al., 2015; Gao et al., 2017). A steeper exponent is indicative of a more inhibition- dominated (lower E/I) state, which has been associated with reduced population spiking and increased scale-free neural activity (Miller et al., 2009; Voytek &Knight, 2015; Zhou &Yu, 2018; Medel et al., 2023; Diachenko et al., 2024). Moreover, in our study, the decreased low-frequency (theta, beta) power in conjunction with a steeper exponent predicts illusory percept is consistent with previous reports that low-frequency power is correlated with population spiking (Miller et al., 2007b, 2009; Ball et al., 2008; Voytek &Knight, 2015), suggesting that reduced synchronous low-frequency activity, in our case, occipital theta and global beta, associated with steeper exponent (less population spiking) may facilitate prestimulus oscillatory dynamics that shapes McGurk illusory perception.

Moreover, we also observed an increase in posterior (occipital and parietal) gamma power predicted the illusory perception when the exponent was steeper. These observations support the notion that gamma activity, often linked to local cortical excitation and perceptual binding, may be more functionally relevant in a subcritical (or inhibition- dominated) state (Cohen &Maunsell, 2011; Harris &Thiele, 2011; Waschke et al., 2021). This interplay between aperiodic and mean power highlights the importance of considering both oscillatory and scale-free neural dynamics in understanding perceptual outcomes. Our interaction model results suggest that a transient shift toward a more inhibited, less spiking activity, and more ordered neural state, indexed by a steeper aperiodic exponent and selective modulation of mean power, may facilitate the perceptual binding processes underlying the McGurk illusion. Overall, we speculate that while different oscillatory features govern distinct cognitive functions (Haegens et al., 2014; Mierau et al., 2017; Scally et al., 2018), they also interact significantly to modulate individual participants' predisposition of prestimulus brain state over individual trials, which leads to experiencing illusory or non-illusory perception in case of McGurk stimulus." (Figure 7) (Figure Legend: Line 1156-1165, Page 51) Visualization of the relationship between prestimulus aperiodic exponent and aperiodic adjusted mean power in predicting McGurk illusory response at: (A) Occipital Theta, (B) Alpha, (C) Beta, and (D) Occipital and Parietal Gamma band power. For (A), (C), and (D) frequency band models, x-axis represents the mean power (aperiodic adjusted) in the frequency range (higher values indicate higher power) and y-axis represents the probability (in percentage) of perceiving the illusion. Note that the distinction of aperiodic exponent into shallow, moderate, and steeper facets are for visualization purposes only, the aperiodic slopes were entered as continuous predictors in all the models. Also, note that (B) illustrates effect of individual predictors: alpha power (left; x- axis, higher values indicate higher power), and exponent (right; x-axis, absolute higher values indicate steeper slope) for prediction to response (y-axis). The shaded region indicates 83% CI. (Minor) 1. "Abstract: The description "frontal-occipital" does not make much sense. I suggest using "frontal and occipital"." Response: Corrected. The sentence now reads as follows: (Line 10-13, Page 1) "Using logistic mixed-effect models, we reveal that the illusory perception is associated with reduced prestimulus alpha (8-12 Hz) and beta (15-30 Hz) power over frontal and occipital regions, increased theta (4-7 Hz) power in parietal and occipital regions, and increased gamma (31-45 Hz) power across the scalp." 2. "Abstract: I suggest splitting the description of the power effects from the associated statistical evidence. The sentence stub "with extreme evidence in the frontal and temporal regions" does not make much sense without a qualifier for what this is extreme. Moreover, please be more precise in the description of the effects: "oscillations" are characterized by power, phase, and frequency. I therefore suggest using "power" here." Response: We thank the reviewer for their suggestion. We agree with the reviewer that the description of power effects and the associated statistical evidence should be clearly separated for better readability and precision. We realized that including the statistical evidence in the abstract does not significantly enhance the main observations being reported. Therefore, we have omitted the line: "with extreme evidence in the frontal and temporal regions (BF > 100)." The revised sentence now states: (Line 10-13, Page 1) "Using logistic mixed-effect models, we reveal that the illusion perception is associated with reduced prestimulus alpha (8-12 Hz) and beta (15-30 Hz) power over frontal and occipital regions, increased theta (4-7 Hz) power in parietal and occipital regions, and increased gamma (31-45 Hz) power across the scalp." 3. "Predicting response to the McGurk trials from prestimulus parameterized power: Thank you for the explanation of the 2639 data points. Unfortunately, this explanation is missing from the paper, so the uninformed reader can't follow this. Moreover, because you performed this analysis separately for the 6 sensor regions, I don't think the information provided in brackets is clear. I suggest including the explanation provided in the rebuttal regarding the trial number instead of this confusing statement." Response: We thank the reviewer for the suggestion and fully agree that the explanation of the 2,639 data points should be clearly presented in the manuscript. In our previous rebuttal, we described these as the number of McGurk trials remaining after EEG preprocessing and behavioral response filtering. While partially correct, this explanation was incomplete. To clarify, the 2,639 data points used in our logistic mixed-effects model do not correspond to full trials but instead represent region-by-trial combinations. Specifically, spectral parameters were extracted from six predefined sensor regions (Frontal, Central, Parietal, Occipital, Left Temporal, Right Temporal) for each McGurk trial. A region-trial pair was included in the analysis only if the SpecParam/ FOOOF model identified valid periodic parameters (center frequency, peak power, and bandwidth) across the four frequency bands (theta, alpha, beta, gamma). Region-trial combinations with missing values in any band were excluded. As a result, the final dataset consisted of a total of 2,639 valid region-trial data points. We regret the earlier lack of clarity and have now addressed this in both the manuscript and the current response. We sincerely appreciate the reviewer's detailed and constructive feedback. We have now revised the Methods section of the manuscript to clearly and transparently describe this structure and inclusion criterion. (Line 281-292, Page 13-14; Materials and Methods section) "Prediction analyses were performed at the level of individual trial-region combinations. Each participant completed 150 McGurk trials, resulting in a potential total of 18×150 = 2,700 trials. Following EEG preprocessing and exclusion of trials with missing or incorrect behavioral responses (responses provided after the stimulus presentation), parameterized spectral features (center frequency, peak power, and bandwidth) were extracted from six anatomically defined sensor regions (Frontal, Central, Parietal, Occipital, Left Temporal, Right Temporal; see Table 1). A region-trial pair was included in the analysis only if valid periodic parameters were identified by the SpecParam/ FOOOF model in all four frequency bands (theta, alpha, beta, gamma). Region-trial pairs with missing values in any frequency band were excluded. This resulted in a total of 2,639 valid region-trial data points used in the logistic mixed-effects models. This stringent criterion ensured that all model predictors were based on reliably detected oscillatory activity (Donoghue et al., 2020b, 2021)." Reference added: (Line 811-813, Page 36) "Donoghue, T., Schaworonkow, N., &Voytek, B. (2021). Methodological considerations for studying neural oscillations. European journal of neuroscience, 55(11-12), 3502-3527. https://doi.org/10.1111/ejn.15361" 4. "Variability in illusory percept observed within and between participants: What does the sentence "the paired sample t test revealed no significance" refer to? I assume you compared the response probability between conditions. If so, I would explicitly add this to the results of the t-test (e.g.: "the paired sample t test revealed no significant difference in the number of illusion and non-illusion trials")." Response: Thank you for the suggestion. However, the behavioral results and Figure 2B (ii) have now been updated based on Reviewer 2's comment #1. Below are the new results updated: (Line 352-372, Page 17-18) "We observed a high degree of inter-individual variability in McGurk susceptibility (Figure 2B (i)). McGurk susceptibility for each participant was quantified as the total percentage of illusory /ta/ responses to the McGurk stimulus. Across the eighteen participants, the percentage of illusory responses ranged widely from 4% to 100% (indicating an illusory percept on every McGurk trial), with a median response of 60.83%. We also calculated the response tendency (inter-trial variability), which is the relative proportion of illusory responses for all the McGurk trials across all eighteen participants (Bechtold &Bastian, 2016). For incongruent McGurk condition, illusion was perceived in 58.38% (SD = 32.5) of trials, whereas unisensory /pa/ (auditory) was perceived in 37.39% (SD = 31.94), and unisensory /ka/ (visual) in 1.68% (SD = 4.97) of total trials presented across all participants. The remaining 2.55% of trials were either responded as "something else" (1.53% of total trials, SD = 2.85) or no response at all (1.02% of trials). To statistically compare response distributions, we performed Friedman's test, which revealed a significant difference among the three response categories (χ²(2) = 23.35, p < 0.001, Kendall's W = 0.649) to McGurk stimulus. Post-hoc pairwise comparisons using the multcompare function in MATLAB with the Tukey- Kramer correction for multiple comparisons, revealed no significance (p = 0.827) between the number of illusion and unisensory /pa/ (auditory) trials. However, significant differences between illusion and unisensory /ka/ (visual) (p < 0.001) and between unisensory /pa/ and unisensory /ka/ (p < 0.001) was observed (see Figure 2B (ii)). Because very few participants reported the visual /ka/ response to the McGurk stimulus, we considered the auditory /pa/ response (17 out of 18 participants) as the non-illusory trial condition for subsequent analyses. This choice allowed for a more reliable within-subject comparison between illusory /ta/ and non- illusory /pa/ perception across trials. Response tendency was also estimated for the congruent trial conditions where participants correctly responded to congruent AV stimuli in 96.54% (SD = 2.66) of trials. We then proceeded with analyzing the electrophysiological data for both illusory and non-illusory McGurk trials." 5. "Periodic and aperiodic oscillations of the whole brain differ between illusory and non- illusory McGurk trials: First, please note that the aperiodic component of neural activity is not an oscillation. I therefore suggest changing the section title. Second, can you please add the degrees of freedom to the report of t-values. This would allow appreciating the number of observations included here. Please also keep the number of decimals and the reporting of effect sizes consistent. Similar, why did you perform non-parametric tests for the aperiodic parameters? This is not described in the methods section. Not finding a significant difference in offset and exponent also does not allow a statement such as "We found lower...". This also does not allow a statement such as "These results imply that there are differences in prestimulus aperiodic activity...". In general, I still don't think it is a valid approach to pool trials across participants here and in the examination of spatial topographies." Response: Below we have responded to each question in a point-by-point fashion:

A. "First, please note that the aperiodic component of neural activity is not an oscillation. I therefore suggest changing the section title." Response: We thank the reviewer for pointing this out. We agree that the aperiodic component of neural activity is not an oscillation. We have now revised the section title as: (Line 379-380, Page 18) "No group-level differences observed in periodic power and aperiodic activity between illusory and non-illusory McGurk trials" B. "Second, can you please add the degrees of freedom to the report of t-values. This would allow appreciating the number of observations included here. Please also keep the number of decimals and the reporting of effect sizes consistent." Response: The results for the following section have been updated based on the Major comment #2. In the current version of the manuscript, the statistical test applied to compare group-level differences between the illusory and non-illusory condition is the repeated-measures ANOVA. However, we have taken into consideration the current recommendations and reported the degrees of freedom and effect sizes (partial eta- squared for repeated measures ANOVA and Cohen's D for post-hoc pairwise comparisons) to the observed values reported. Additionally, we have ensured consistency in the number of decimals and the reporting of effect sizes throughout the text: (Line 377-410, Page 18-19) "A repeated measures ANOVA with a 2 (Condition: /ta/ and /pa/) x 4 (Frequency: Theta, Alpha, Beta, Gamma) within-subjects design was computed to analyse power spectrum differences (PSDs) between illusory and non-illusory response conditions. During prestimulus duration, the analysis revealed no significant interaction between Condition and Frequency (F(3,48) = 0.239, pGG = 0.868, partial η2 = 0.015). However, post-hoc pairwise comparison with Tukey-HSD multiple correction revealed significantly higher beta power (mean difference = 0.339, 95% CI = [0.015, 0.662], p= 0.041, Cohen's D = 0.539) and higher gamma power (mean difference = 0.575, 95% CI = [0.229, 0.921], p = 0.003, Cohen's D = 0.854) before illusory perception (Figure 3A (a)). For poststimulus duration, the repeated measures ANOVA revealed no significant interaction between Condition and Frequency (F(3,48) = 0.529, pGG = 0.571, partial η2 = 0.032). Post-hoc tests revealed no significant condition differences across frequency bands (Figure 3A (b)). Consistent with previous findings by Keil et al. (2012), we observed higher prestimulus beta-band power associated with illusory perception; however, we did not observe a reduction in theta power following illusory perception.

We further examined the group-level differences in periodic power and aperiodic component estimated by parameterizing the power spectrum using SpecParam (FOOOF) model (Donoghue et al., 2020b). The periodic oscillations were computed after removing the aperiodic component in linear space from untransformed power spectrum from trials where peaks were detected after fitting the FOOOF model. A repeated measures ANOVA with a 2 (Condition: /ta/ and /pa/) x 6 (Component: mean theta, alpha, beta, gamma power; aperiodic offset, aperiodic exponent) within-subjects design was computed to analyse differences in periodic power and aperiodic component between illusory and non-illusory response conditions. We observed no significant interaction between Condition and Component for either the prestimulus (F(5,80) = 0.343, pGG = 0.632, partial η2 = 0.021; Figure 3B) or poststimulus duration (F(5,80) = 1.548, pGG = 0.227, partial η2 = 0.09; Extended Data Figure 3-1). Moreover, post-hoc tests also revealed no significant pairwise condition difference for aperiodic components (offset and exponent) and periodic power across frequency bands. These findings emphasize the importance of separating aperiodic activity from periodic oscillations to accurately characterize the spectral changes associated with the McGurk illusory percept. Importantly, the absence of group-level effects prior to McGurk perception suggests that individual differences in susceptibility may obscure perception-related neural dynamics, making inter-trial analyses a more sensitive approach to uncover the underlying mechanisms before McGurk perception. We then proceeded to capture the trial-wise variability at the sensor space." C. "Similar, why did you perform non-parametric tests for the aperiodic parameters? This is not described in the methods section. Not finding a significant difference in offset and exponent also does not allow a statement such as "We found lower...". This also does not allow a statement such as "These results imply that there are differences in prestimulus aperiodic activity...". In general, I still don't think it is a valid approach to pool trials across participants here and in the examination of spatial topographies." Response: Thank you for raising this crucial point regarding the unpaired tests performed in the previous version of the manuscript. We realize the gravity of running into Type I error in group- level statistics by not accounting for inter-subject variability. So based on your Major comment #2, we have revised the group-level statistics by applying repeated measures ANOVA. Below is the excerpt from the manuscript discussing about how we computed the ANOVA model for computing differences in periodic power and aperiodic activity between illusory and non-illusory conditions at the inter-individual level. (Line 209-225, Page 10-11; Materials and Methods section) At the group-level "To examine group-level differences in spectral power (both prestimulus and poststimulus) between illusory (/ta/) and non-illusory (/pa/) trials across frequency bands, a repeated- measures analysis of variance (ANOVA) was performed using MATLAB's ranova function. The analysis employed a 2 (Condition: /ta/, /pa/) x 4 (Frequency: Theta, Alpha, Beta, Gamma) within-subject design. For each participant, mean power values were computed for each frequency bands and condition, and these were fit into the repeated measures ANOVA model. One participant with 100% illusory /ta/ McGurk susceptibility was excluded from the group- level pairwise analysis. Sphericity was evaluated using Mauchly's test, and Greenhouse- Geisser corrections for p-values (pGG) were applied when the assumption of sphericity was violated. Partial eta-squared (η2) was calculated to estimate effect sizes for main and interaction effects. Post-hoc pairwise comparisons between condition within each frequency band were performed using Tukey HSD test for multiple comparison corrections. To further dissociate condition effects on periodic power and aperiodic components, a similar repeated-measures ANOVA was conducted on parameterized spectral features. This analysis used a 2 (Condition: /ta/, /pa/) × 6 (Component: mean theta, alpha, beta, gamma power; aperiodic offset, aperiodic exponent) within-subjects design. The same procedures for sphericity assessment, correction, effect size estimation, and post-hoc testing were applied as in the power spectral analysis." (Line 393-410, Page 18-19; Results section) "We further examined the group-level differences in periodic power and aperiodic component estimated by parameterizing the power spectrum using SpecParam (FOOOF) model (Donoghue et al., 2020b). The periodic oscillations were computed after removing the aperiodic component in linear space from untransformed power spectrum from trials where peaks were detected after fitting the FOOOF model. A repeated measures ANOVA with a 2 (Condition: /ta/ and /pa/) x 6 (Component: mean theta, alpha, beta, gamma power; aperiodic offset, aperiodic exponent) within-subjects design was computed to analyse differences in periodic power and aperiodic component between illusory and non-illusory response conditions. We observed no significant interaction between Condition and Component for either the prestimulus (F(5,80) = 0.343, pGG = 0.632, partial η2 = 0.021; Figure 3B) or poststimulus duration (F(5,80) = 1.548, pGG = 0.227, partial η2 = 0.090; Extended Data Figure 3-1). Moreover, post-hoc tests also revealed no significant pairwise condition difference for aperiodic components (offset and exponent) and periodic power across frequency bands. These findings emphasize the importance of separating aperiodic activity from periodic oscillations to accurately characterize the spectral changes associated with the McGurk illusory percept. Importantly, the absence of group-level effects prior to McGurk perception suggests that individual differences in susceptibility may obscure perception-related neural dynamics, making inter-trial analyses a more sensitive approach to uncover the underlying mechanisms before McGurk perception. We then proceeded to capture the trial-wise variability at the sensor space." 6. "Prestimulus periodic and aperiodic parameters across different sensor regions can predict the response to the upcoming McGurk stimulus: A statement such as "Aperiodic offset and exponent parameters from central, parietal, and occipital regions were found to be better predictors to predict response to upcoming McGurk stimulus" would require stating a comparison (i.e., "better" than what) and a statistical test to qualify such a comparison." Response: We thank the reviewer for pointing out the ambiguity in the observations that we had earlier reported. We have now revised the sentence as follows: (Line 451-455, Page 21) "Aperiodic offset and exponent parameters from central and parietal sensors showed extreme evidence (BF > 100) for predicting the response to the upcoming McGurk stimulus. Whereas aperiodic offset from occipital sensors showed strong evidence (10 < BF < 100), and occipital exponent showed moderate evidence (3 < BF < 10) in predicting illusory responses." Reviewer 2 1. "-Interpretation This point is crucial. According to my understanding, the discussion and results are inconsistent. In the previous review I asked the authors to check for a bias towards the visual modality, as they claimed, or to remove this claim from the discussion. The authors chose to reformulate the statement in the discussion, and I wonder why they did not perform a simple t-test to check whether subjects displayed a preference towards visual stimulus in the incongruent non-illusory trials. More importantly, this statement does not fit with what is reported in the paper I will copy below the paragraphs that make consider there is an inconsistency in the results. In the Methods section, the authors write: "Each participant viewed 600 trials of videos of a male annotator (matched by the geographic region and demographic background of the participants) articulating the syllables /pa/, /ta/, and /ka/. Participants were instructed to report their subjective perceptions of the audio-visual (AV) stimuli. The experiment included four types of AV stimuli: three congruent (audio syllables matching video articulation) syllables /pa/, /ta/, and /ka/, and one McGurk (audio-visual mismatch) syllable (auditory /pa/ with visual /ka/), which created the illusion of syllable /ta/." So, according to this paragraph, /pa/ corresponds to the auditory part of the McGurk stimulus. Then, behavioural results are reported (Results section): "For incongruent McGurk condition, illusion was perceived in 58.38% (SD = 32.5) of trials, whereas, unisensory /pa/ was perceived in 37.39% (SD = 31.94) of trials (Figure 2B (ii))." For clarity, the modality of /pa/ should be stated. According to the methods, /pa/ is auditory. Therefore, the results describe that in the incongruent trials 58.38% are illusory, 37.39% are perceived as the auditory modality (/pa/), which would leave 4.23% trials perceived as the visual modality.

Finally, in the discussion, the authors talk about a possible bias towards the visual sensory input: "While we did not directly measure behavioral indices of visual bias in our participants, these studies, however, suggests that the lower prestimulus alpha observed in our study (Figure 3A) specifically in the occipital sensors (Figure 4) prior to the McGurk illusion might reflect a perceptual bias towards visual sensory input." Given the numbers reported in the behavioral section, if there is a bias, wouldn't the authors interpret, according to the data they report, that the bias would be towards the auditory modality? This needs to be clarified. As it is clear from the methods that the authors have the data required to test for a potential bias (preferred modality in the non-illusory incongruent trials), they should do the test or remove the paragraph from the discussion." Response: We thank the reviewer for highlighting this important inconsistency between the results and discussion section. We agree that given our experimental design, participants have responded either auditory (/pa/), visual (/ka/), or illusory (/ta/) responses for the incongruent McGurk trials, and that a statistical comparison of these three proportions is needed to support or withdraw our claim of a visual or auditory bias. We have updated the behavioral results section as well as the Discussion section to incorporate these observations: (Line 358-372, Page 17; Results section) "For incongruent McGurk condition, illusion was perceived in 58.38% (SD = 32.5) of trials, whereas, unisensory /pa/ (auditory) was perceived in 37.39% (SD = 31.94), and unisensory /ka/ (visual) in 1.68% (SD = 4.97) of total trials presented across all participants. The remaining 2.55% of trials were either responded as "something else" (1.53% of total trials, SD = 2.85) or no response at all (1.02% of trials). To statistically compare response distributions, we performed non-parametric Friedman's test, which revealed a significant difference among the three response categories (χ²(2) = 23.35, p < 0.001, Kendall's W = 0.649) to McGurk stimulus. Post-hoc pairwise comparisons using the multcompare function in MATLAB with the Tukey-HSD correction for multiple comparisons, revealed no significance (p = 0.827) between the number of illusion and unisensory /pa/ (auditory) trials. However, significant differences between illusion and unisensory /ka/ (visual) (p < 0.001) and between unisensory /pa/ and unisensory /ka/ (p < 0.001) was observed (see Figure 2B (ii)). Because very few participants (8 out of 18 participants) reported the visual /ka/ response to the McGurk stimulus, we considered the auditory /pa/ response (17 out of 18 participants) as the non-illusory trial condition for subsequent analyses. This choice allowed for a more reliable within-subject comparison between illusory /ta/ and non-illusory /pa/ perception across trials." (Figure Legend - Line 1116-1119, Page 49) "(ii) Violin plot showing inter-trial variability - percentage of /ta/ (illusory), unisensory /pa/ (auditory), and unisensory /ka/ (visual) percept during the presentation of McGurk stimulus and the congruent AV stimuli (/pa/, /ta/, and /ka/). The white dot in the center of each violin plot represents the median. Significance levels are denoted by asterisks: '****' p {less than or equal to} 0.0001, and 'n.s' is not-significant." (Figure 2B) (Line 591-611, Page 27-28; Discussion section) "Moreover, lower prestimulus alpha band power has been associated with higher cortical excitability (Mathewson et al., 2009, 2011; Romei et al., 2008, 2010; Samaha et al., 2017). This means that at regions with high excitability (lower alpha power), the threshold of activation of the underlying neural population is lowered. Thus, improving the neural processing of sensory input leading to better perception, which in case of incongruent McGurk stimulus sometimes may lead to illusory percept (Van Dijk et al., 2008; Jensen et al., 2012; Lange et al., 2014). In our study, we observed lower prestimulus alpha periodic power in the frontal and central sensor regions prior to illusory perception (Figure 4B). This might reflect increased excitability or attentional engagement of higher-order networks that might be critical for multisensory integration in McGurk illusion (Foxe &Snyder, 2011; Haegens et al., 2012; Sadaghiani &Kleinschmidt, 2016). Fluctuations in frontal and central alpha power have also been linked to top-down modulation of attention and preparatory states that influence perceptual outcomes (Jones et al., 2010; Klimesch, 2012; Samaha et al., 2015a). Notably, while we did not find significant occipital alpha power differences at the sensor level, our analysis of peak alpha frequency (PAF) in the occipital region (Figure 5E) revealed that lower PAF predicted illusory percepts that is consistent with previous findings that occipital PAF modulates audiovisual integration and susceptibility to multisensory illusions (Cecere et al., 2015; Keil &Senkowski, 2017; Samaha &Postle, 2015b; Venskus &Hughes, 2021). Taken together, these results suggest that lower prestimulus alpha power in frontal and central regions, along with lower occipital PAF, may jointly facilitate the attentional and integrative processes that bias perception toward the McGurk illusion. This aligns with the view that alpha oscillations reflect both local excitability and large-scale network dynamics that shape multisensory perceptual experiences (Foxe &Snyder, 2011; Sadaghiani &Kleinschmidt, 2016)." 2. "-Background The authors have now improved the introduction, adding information about aperiodic activity and the clarifying the relevance of their approach for the study of McGurk illusion. However, there are still some gaps that need to be filled.

The authors seem to assume that brain oscillations and aperiodic activity result from different neural processes. This is a valid hypothesis, but still they should acknowledge that there is an alternative work hypothesis: that aperiodic activity and brain oscillations coexist and can result from same neural populations. See, for instance: Gireesh, E.

D. &Plenz, D. Neuronal avalanches organize as nested theta- and beta/gamma- oscillations during development of cortical layer 2/3. Proceedings of the National Academy of Sciences 105, 7576-7581 (2008). The justification for including bandwidth provided in the introduction is feeble, just because a parameter has not been explored, this does not mean that is relevant. However, in the discussion the authors do provide an interpretation that justifies the interest of evaluating bandwidth. I suggest that the authors mention the review of Lowett et al (2022) already in the introduction to strengthen the motivation of including bandwidth in the analyses." Response: We thank the reviewer for highlighting these important points and for the suggestion to broaden the theoretical background of our work. In the revised manuscript, we have addressed both concerns:

A. First, we now acknowledge the alternative hypothesis that aperiodic activity and brain oscillations may coexist and emerge from the same neural populations, as shown by Gireesh and Plenz (2008), and we have cited this important study in the introduction: (Line 62-73, Page 4) "While these studies have considered oscillatory and aperiodic activity as arising from distinct neural mechanisms, alternative studies suggest that these two components might be originating from the same underlying neuronal population dynamics. For instance, Gireesh &Plenz (2008) demonstrated that neuronal avalanches in developing cortical networks organize as nested theta and beta/gamma oscillations, indicating a potential co-generation of rhythmic and arrhythmic activity. Similarly, Haegens et al., (2011), reported that alpha (8-14 Hz) oscillatory activity is negatively correlated with the spiking rates of underlying neuronal populations, especially in the somatosensory cortex, further underscoring this interdependence. Together, these studies highlight that the two components of the power spectrum: oscillations and aperiodic activity whether originating from the shared or distinct neural mechanisms may represent complementary aspects of brain dynamics, and the balance between these two components might affect cognitive processes." Reference added (Line 839-841, Page 38):

Gireesh, E. D., &Plenz, D. (2008). Neuronal avalanches organize as nested theta-and beta/gamma-oscillations during development of cortical layer 2/3. Proceedings of the National Academy of Sciences, 105(21), 7576-7581. https://doi.org/10.1073/pnas.0800537105 B. Second, we have provided the rationale for including bandwidth as a parameter by referencing the review by Lowet et al., (2022) and a few more references like Cole &Voytek, (2019); and, Donoghue et al., (2020b), and a recent work by Thuwal et al., (2021) earlier in the introduction. (Line 79-83, Page 4) "Although, the role of bandwidth remains underexplored, studies suggest that bandwidth parameter along with center frequency offer valuable insights into neural synchrony and circuit dynamics (Cole &Voytek, 2019; Donoghue et al., 2020b). A recent work by Thuwal et al., 2021 and a review by Lowet et al., (2022) suggests that variability in oscillatory parameters might reflect changes in neural computations across different cognitive tasks." 3. "-Multiple comparison correction, I am surprised that the p-values after multiple comparison correction remain completely unchanged. Could the authors explain how this was performed? I have the impression that they applied the correction separately for each model, am I right? I believe I was not clear in my previous review, but I would be more in the line of correcting for the different tests performed (for instance, different frequency bands evaluated)." Response: We appreciate the reviewer's concern regarding the multiple comparison correction and acknowledge that our previous description may have lacked sufficient detail. To clarify, in the initial submission we applied the Benjamini-Hochberg False Discovery Rate (FDR) correction separately within each set of predictors across models, such as frequency-specific logistic mixed interaction models and region-specific logistic mixed-models. This group-wise correction is a standard approach, particularly when predictors within each model are thought to be conceptually distinct. However, we understand the reviewer's viewpoint for a more conservative correction across all tests to reduce any false positive effects seen across models in our study, since the predictors cannot for sure be considered completely independent. In line with this, we have now re- analysed the data using a global FDR correction that accounts for all predictors across all frequency bands models and all sensor region interaction models. This change has been reflected in the revised manuscript, and we confirm that all key results remain statistically significant under this more stringent correction (see Table 2 and Extended Data Figure 5-1 to 5-7). The Methods section has also been updated to explicitly describe this revised procedure. We thank the reviewer for encouraging us to enhance the clarity and statistical rigor of our analysis. (Line 260-262, Page 12) "To control for multiple comparisons across all frequency models, p-values of all the model predictors were adjusted using the Benjamini-Hochberg method (Benjamini &Hochberg, 1995), maintaining the false discovery rate (FDR) at α = 0.05." (Line 344-345, Page 16) "To control for multiple comparisons, p-values obtained from the post-hoc tests were adjusted using Benjamini-Hochberg method across all sensor region models." 4. "-Parametrized power The authors claim that they separate aperiodic power from oscillatory activity, but this is not completely true. The authors do not check, according to the report, whether the activity that remains in a given frequency band corresponds to an oscillation or not (See, for instance: https://onlinelibrary.wiley.com/doi/10.1111/ejn.15829). Even if aperiodic contribution is removed from the data, this does not imply that the remaining activity is periodic. It would be important that the authors acknowledged this limitation in their study and the implications this could have for the interpretation of the results. For instance, according to Figure 3 and Extended data figure 3.1 there is no peak in theta activity (either in parametrized and non- parametrized power), and therefore the differences observed in theta could be due to shifts in alpha peak, or changes in the bandwidth of alpha, as one could interpret from the graph." Response: We thank the reviewer for this insightful comment. We agree that by subtracting the aperiodic component from the power spectrum does not inherently guarantee that the remaining activity reflects true oscillations, as residual power in canonical bands could arise from non-oscillatory spectral features (e.g., overlapping peaks). We would like to point out that study by Donoghue et al., (2020b) suggest that periodic oscillations appear as distinct spectral peak above the aperiodic fit. In our study, we applied the SpecParam/ FOOOF algorithm to fit channel-wise Gaussian peaks on trial-by-trial basis across four canonical bands (theta: 4-7 Hz; alpha: 8-12 Hz; beta: 15-30 Hz; gamma: 31-45 Hz). Only peaks exceeding the aperiodic background were retained as oscillatory features. We would also like to point out that Figure 3 and Extended Data Figure 3-1 showcases the grand- averaged data (channel and trial-averaged across participants) for the original untransformed power spectrum and periodic power. This averaging process may have obscured narrowband theta peaks, as these peaks were not maximally present in all the channels. Moreover, we restricted our logistic mixed-effects models to region-trial combinations in which valid peaks were detected across all four frequency bands, ensuring that theta peak parameter was derived from a trial where a distinct oscillatory peak was identified, minimizing the risk of broadband or residual 1/f artifacts driving our results. While this conservative approach greatly reduces the risk that broadband or 1/f artifacts drive our findings, we acknowledge that occasional overlap with adjacent alpha activity cannot be ruled out entirely. Accordingly, we have updated both the Methods and Discussion section of the manuscript to explicitly acknowledge these caveats as a limitation in our study and to clarify that we did not assume every residual component is periodic unless supported by a fitted spectral peak. (Line 282-293, Page 13-14: Materials and Methods section) "Prediction analyses were performed at the level of individual trial-region combinations. Each participant completed 150 McGurk trials, resulting in a potential total of 18×150 = 2,700 trials. Following EEG preprocessing and exclusion of trials with missing or incorrect behavioral responses (responses provided after the stimulus presentation), parameterized spectral features (center frequency, peak power, and bandwidth) were extracted from six anatomically defined sensor regions (Frontal, Central, Parietal, Occipital, Left Temporal, Right Temporal). A region-trial pair was included in the analysis only if valid periodic parameters were identified by the SpecParam/ FOOOF model in all four frequency bands (theta, alpha, beta, gamma). Region-trial pairs with missing values in any frequency band were excluded. This resulted in a total of 2,639 valid region-trial data points used in the logistic mixed-effects models. This stringent criterion ensured that all model predictors were based on reliably detected oscillatory activity (Donoghue et al., 2020b, 2021)." (Line 580-589, Page 27; Discussion section) "However, a key caveat to consider with narrowband theta peaks, is that the peak differences observed might stem from non-oscillatory spectral features (Donoghue et al., 2021), or shifts in neighboring bands (changes in alpha peak frequency or bandwidth) (see Seymour et al., 2022). Although we enforced stringent inclusion criteria so that only trials with valid periodic parameters in all four canonical bands were modelled, we cannot entirely rule out subtle cross-band interactions or transient non-oscillatory events contributing to our results. Future work that combines spectral parameterization with time- domain burst detection (Wen &Liu, 2016; Seymour et al., 2022; Wilson et al., 2022) or phase-based analyses (like cycle-by-cycle methods; see Cole &Voytek 2019) will help to further disambiguate true oscillations from spectral leakage or aperiodic fluctuations." References added: (Line 811-816, Page 36) "Donoghue, T., Schaworonkow, N., &Voytek, B. (2021). Methodological considerations for studying neural oscillations. European journal of neuroscience, 55(11-12), 3502-3527. https://doi.org/10.1111/ejn.15361" (Line 1034-1036, Page 45) "Seymour, R. A., Alexander, N., &Maguire, E. A. (2022). Robust estimation of 1/f activity improves oscillatory burst detection. European Journal of Neuroscience, 56(10), 5836- 5852. https://doi.org/10.1111/ejn.15829" (Line 1075-1076, Page 47) "Wilson, L. E., da Silva Castanheira, J., &Baillet, S. (2022). Time-resolved parameterization of aperiodic and periodic brain activity. Elife, 11, e77348. https://doi.org/10.7554/eLife.77348" 5. "-Citation: Following citation:

Fernandez M, L., Torralba, M., &Soto‐Faraco, S. (2018). Theta oscillations reflect conflict processing in the perception of the McGurk illusion. European Journal of Neuroscience, 48(7), 2630-2641. https://doi.org/10.1111/ejn.13804 Should be Morís-Fernández L., Torralba, M., &Soto‐Faraco, S. (2018). Theta oscillations reflect conflict processing in the perception of the McGurk illusion. European Journal of Neuroscience, 48(7), 2630-2641. https://doi.org/10.1111/ejn.13804" Response: Citation updated. We thank reviewer for the suggestion.

6. "-Data availability statement The authors need to provide a valid link to the data and code for the review process: "The raw EEG data used in this study are available from the corresponding authors upon reasonable request. However, the processed EEG data and all the relevant codes used for subsequent analysis are available in the provided link (URL is redacted)"." Response: Adhering to the double-blind review guideline of the journal, we initially redacted the URL for the processed EEG data and relevant codes. However, we realized that we can anonymously share the data on the OSF (Open Science Framework) platform, so we have updated the URL link with the authors' names anonymized in the manuscript: (Line 743-748, Page 34) Data Availability Statement "The raw EEG data used in this study were reanalyzed from a previous study published by Kumar et al., 2020. Therefore, access to the raw EEG data can be obtained by contacting the corresponding authors of the original study. However, the processed EEG data and all the relevant codes used in this study are available in the provided link: https://osf.io/degzm/?view_only=8cc46c126b36445a8d37c3f6e0f07013"
